# STING-adjuvanted outer membrane vesicle nanoparticle vaccine against *Pseudomonas aeruginosa*

**DOI:** 10.1172/jci.insight.188105

**Published:** 2025-07-24

**Authors:** Elisabet Bjånes, Nishta Krishnan, Truman Koh, Anh T.P. Ngo, Jason Cole, Joshua Olson, Ingrid Cornax, Chih-Ho Chen, Natalie Chavarria, Samira Dahesh, Shawn M. Hannah, Alexandra Stream, Jiaqi Amber Zhang, Hervé Besançon, Daniel Sun, Siri Yendluri, Sydney Morrill, Jiarong Zhou, Animesh Mohapatra, Ronnie H. Fang, Victor Nizet

**Affiliations:** 1 Division of Host-Microbe Systems and Therapeutics, Department of Pediatrics,; 2 Aiiso Yufeng Li Family Department of Chemical and Nano Engineering, and; 3 Skaggs School of Pharmacy and Pharmaceutical Sciences, UCSD, La Jolla, California, USA.

**Keywords:** Infectious disease, Microbiology, Vaccines, Bacterial infections, Bacterial vaccines, Nanotechnology

## Abstract

Multidrug-resistant (MDR) bacterial pneumonia poses a critical threat to global public health. The opportunistic Gram-negative pathogen *Pseudomonas aeruginosa* is a leading cause of nosocomial-associated pneumonia, and an effective vaccine could protect vulnerable populations, including the elderly, immunocompromised, and those with chronic respiratory diseases. Highly heterogeneous outer membrane vesicles (OMVs), shed from Gram-negative bacteria, are studded with immunogenic lipids, proteins, and virulence factors. To overcome limitations in OMV stability and consistency, we described what we believe to be a novel vaccine platform that combines immunogenic OMVs with precision nanotechnology — creating a bacterial cellular nanoparticle (CNP) vaccine candidate, termed Pa-STING CNP, which incorporates an adjuvanted core that activates the STING (stimulator of interferon genes) pathway. In this design, OMVs are coated onto the surface of self-adjuvanted STING nanocores. Pa-STING CNP vaccination induced substantial antigen presenting cell recruitment and activation in draining lymph nodes, robust anti-*Pseudomonas* antibody responses, and provided protection against lethal challenge with the hypervirulent clinical *P*. *aeruginosa* isolate PA14. Antibody responses mediated this protection and provided passive immunity against the heterologous *P*. *aeruginosa* strain PA01. These findings provided evidence that nanotechnology can be used to create a highly efficacious vaccine platform against high priority MDR pathogens such as *P*. *aeruginosa*.

## Abstract 2

An engineered nanoparticle vaccine coated by outer membrane vesicles of the bacterial pathogen *Pseudomonas aeruginosa* elicits strong antibody responses and protects mice against lethal pneumonia

## Graphical abstract

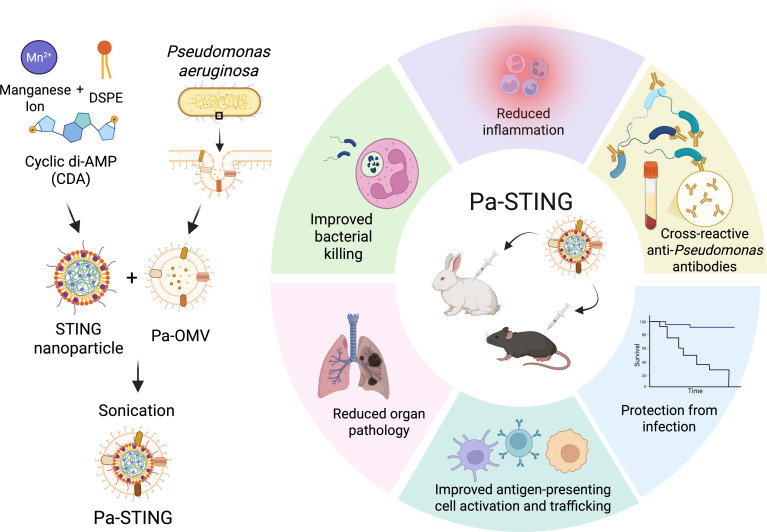

## Introduction

Pneumonia remains a leading cause of global morbidity and mortality, accounting for over 2 million deaths in 2021 (1). The disease poses a particularly high risk to vulnerable populations, including the elderly, immunocompromised, hospitalized individuals, and children (2). Hospital-acquired pneumonia (HAP), defined as a lower respiratory tract infection acquired more than 48 hours after hospital admission, is associated with increased risk of mortality (3). Before the COVID-19 pandemic, 10%–20% of individuals on mechanical ventilation developed ventilator-associated pneumonia (VAP) (3). During the pandemic, VAP rates skyrocketed to 48%–80%, substantially contributing to morbidity and mortality (4, 5). HAP and VAP are notoriously difficult to treat, with mortality rates reaching 40%, largely due to poor antibiotic penetration into lung tissues and the high prevalence of multidrug-resistant (MDR) pathogens (6, 7). While the pool of effective antimicrobials is dwindling, advances in vaccine technology present promising alternatives (8). In particular, the application of nanotechnology to vaccine platforms offers advantages of increased precision, stability, target delivery, and protection from degradation (9–11). The ability to tailor nanoparticle size to optimize uptake by antigen-presenting cells (APCs) is a key advantage of this technology (12–14).

Outer membrane vesicles (OMVs) are small, spherical, bilayered nanostructures naturally shed from Gram-negative bacteria (15). OMVs play various biological roles, including virulence (16, 17), evasion of host immunity (18, 19), gene transfer (20), nutrient acquisition (21), antibiotic resistance (22), and stress responses (23). OMVs have been investigated as vaccine candidates against several pathogens due to their membrane surface composition, which includes highly antigenic lipids, proteins, and virulence factors (24–26). For example, the *Neisseria meningitidis* vaccine Bexsero, licensed for human use, includes OMVs from serogroup B as one of its components (27). Additionally, *Haemophilus influenzae* B (HiB) vaccines use *N*. *meningitidis–*engineered OMVs as a delivery platform for HiB antigens (28). However, the inherent heterogeneity in OMV stability (29), internal cargo (30, 31), size (32), and yield complicates the development of OMV-based vaccines (33). Our previous research demonstrated that applying nanotechnology to OMV-based vaccines could stabilize a cellular membrane on a nanoparticle, enhancing their consistency, stability, and efficacy (34, 35).

We have developed what we believe to be a novel adjuvanted vaccine candidate that harnesses the immunogenic potential of OMVs combined with the stability and precision conferred by an adjuvanted nanoparticle core to create a bacterial cellular nanoparticle (CNP) vaccine platform. For these studies, we selected *Pseudomonas aeruginosa*, an opportunistic Gram-negative pathogen and a leading cause of HAP and VAP (36, 37). MDR and extremely-drug resistant (XDR) *P*. *aeruginosa* strains account for 15%–30% of infections in certain regions, designating it as a World Health Organization high-priority pathogen (38). In addition to pneumonia, *P*. *aeruginosa* is a serious cause of catheter-associated urinary tract infections, secondary burn wound infections, bacteremia, and surgical site infections (39). Historically, 60%–80% of patients with cystic fibrosis (CF) have been colonized with *P*. *aeruginosa,* contributing to disease-related morbidity and mortality (40). While there has been a substantial decline in *P*. *aeruginosa* colonization among CF patients since the introduction of highly effective modulator therapy (HEMT) — a combination of elexacaftor, tezacaftor, and ivacaftor (41, 42) — effective antibiotics against MDR *P*. *aeruginosa* are dwindling, and no vaccines have been approved. A protective vaccine could provide a crucial defense for high-risk individuals without further driving antibiotic resistance.

In this study, we engineered a *P*. *aeruginosa* vaccine candidate using a stimulator of interferon genes–adjuvanted (STING-adjuvanted) nanoparticle core coated with OMVs isolated from a hypervirulent clinical isolate (Pa-STING CNP). The STING pathway is critical for mammalian innate immunity, detecting foreign nucleic acids and upregulating type I IFN signaling, which supports the activation of key antimicrobial defenses (43). STING agonists have recently been explored as cancer metallotherapy (44) and as cancer vaccine adjuvants (45). Notably, the STING pathway is essential for host protection against *P*. *aeruginosa* pneumonia in murine infection models, helping to mitigate excessive inflammatory cytokine production (46, 47).

We report that the Pa-STING-CNP vaccine induces superior APC recruitment and activation in lymph nodes compared with red blood cell–coated STING (RBC-STING) and nonadjuvanted controls. Highly immunogenic, Pa-STING-CNP vaccination elicited robust anti*-P*. *aeruginosa* IgG titers at doses as low as 0.01 μg, providing strong protection against lethal pneumonia challenge. Integrating a STING-adjuvanted core into an OMV-based CNP vaccine may offer an effective and versatile platform for combating MDR pathogens.

## Results

### Pa-STING nanoparticle characterization.

Building upon our previous work, we coated gold nanoparticles (Au-NPs) with OMVs derived from the well-characterized, hypervirulent *P*. *aeruginosa* clinical isolate PA14, following established protocols (34, 35) (see Methods for details). Mice were vaccinated with 3 doses of Pa-NPs at 0.01, 0.1, or 1 μg per dose, or with 1 μg RBC-NPs as a control (Supplemental Figure 1A; supplemental material available online with this article; https://doi.org/10.1172/jci.insight.188105DS1). On day 28, mice were challenged intratracheally with approximately 1 × 10^7^ colony forming units (CFUs) of *P*. *aeruginosa* PA14 — a dose that is lethal in approximately 90% of unvaccinated animals. Vaccination with 1 μg Pa-NP conferred 50% protection compared with RBC-NP controls and elicited modest increases in anti-Pa IgG titers (Supplemental Figure 1, B and C). These findings prompted us to explore the incorporation of a STING adjuvant to enhance the immunogenicity and protective efficacy of the Pa-NP vaccine platform.

**Figure 1:**
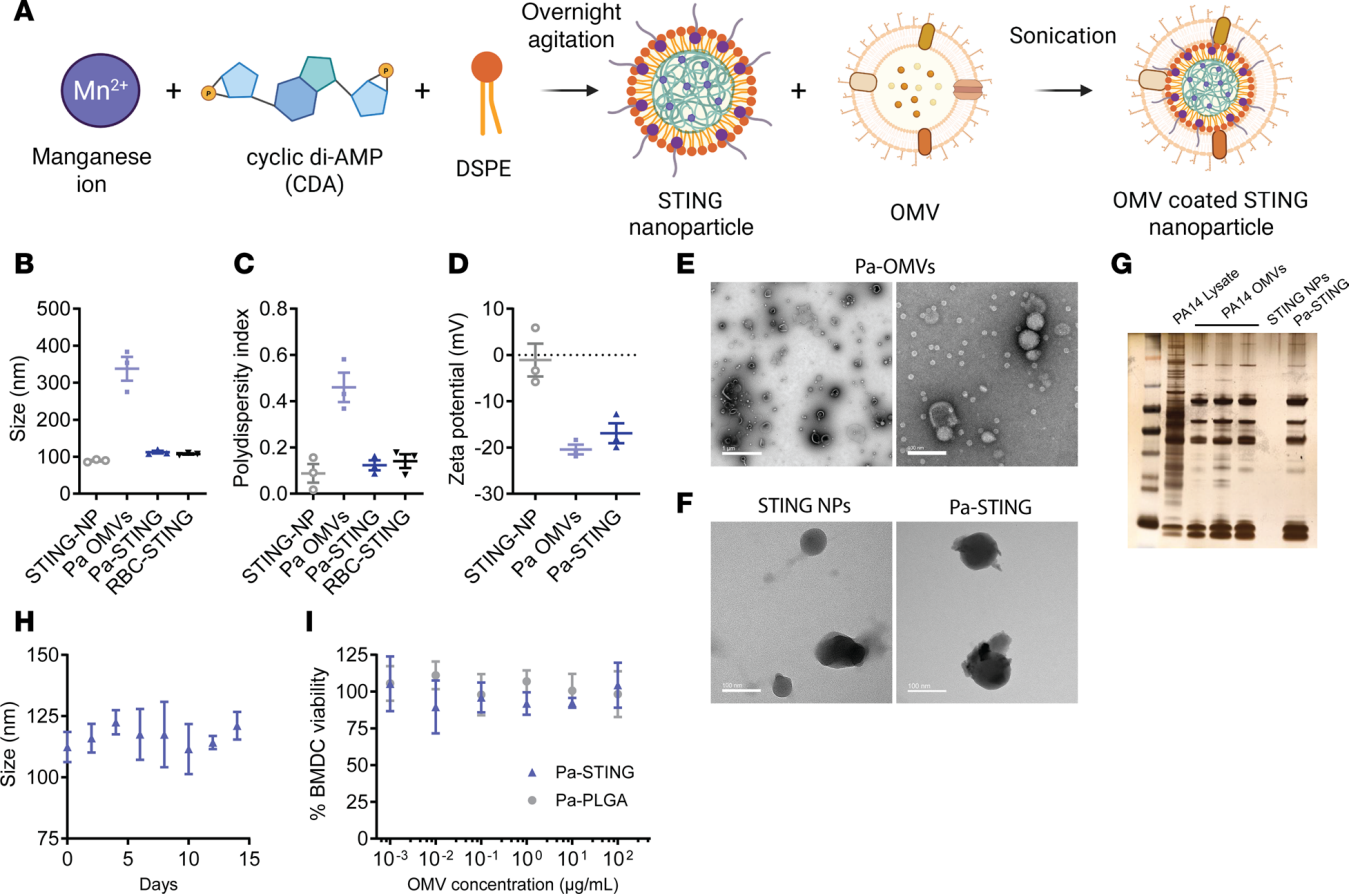
Characterization of Pa-STING NPs. (**A**) STING nanoparticle synthesis. Manganese, cyclic di-AMP (CDA), and DSPE-H11 were combined with overnight agitation to self assemble into STING nanoparticles (NPs). OMV membranes were coated onto the surface of STING NPs by sonication. (**B**) Size distribution, (**C**) polydispersity index, and (**D**) zeta potential of STING-NPs, Pa-OMVs, Pa-STING, and red blood cell (RBC-STING) nanoparticles, as measured by dynamic light scattering (DLS). (**E**) Transmission electron microscopy (TEM) images of negatively stained Pa-OMVs. Scale bars: 1 μm (left), 100 nm (right). (**F**) TEM images of unstained STING NPs and Pa-STING. Scale bar: 100 nm. (**G**) Protein loading of Pa-STING nanoparticles compared to PA14 whole cell lysate, STING NPs and Pa-OMVs. 10 μg of protein or NP measured by BCA assay was loaded per lane and visualized with silver stain. (**H**) Stability of Pa-STING nanoparticles over 2 weeks as measured by DLS. (**I**) Percentage bone marrow–derived dendritic cells (BMDC) viability after incubation with Pa-STING or Pa-PLGA particles for 48 hours. BMDCs were incubated with increasing concentrations of Pa-STING or Pa-PLGA particles and viability was measured by PrestoBlue. Representative of 3 independent experiments.

We engineered the STING-adjuvanted nanoparticle cores from manganese, cyclic di-AMP, and DSPE-H11, a modified phospholipid (44) and then coated with OMVs isolated from PA14, following established protocols (34, 35) (Figure 1A, see Methods for details). A key advantage of using OMV-coated bacterial CNPs over OMVs alone is the inherent adjuvant capacity of the STING core and the ability to precisely tailor CNP size to optimize uptake by APCs, which directs immune responses. We synthesized STING cores at approximately 100 nm, with a slight increase in size observed after OMV coating, as measured by dynamic light scattering (DLS) (Figure 1B). Mouse RBC-STING was used as a membrane control. Consistent with our previous work and the literature, DLS revealed high heterogeneity in the size distribution of PA14 OMVs (Figure 1, B and C). In contrast, the Pa-STING CNPs were uniform in size. Successful coating was confirmed by size and ζ potential measurements, which showed a decrease in charge after coating (Figure 1D). Transmission electron microscopy (TEM) further confirmed successful OMV coating of the nanoparticle cores and illustrated the high morphological heterogeneity of native OMVs (Figure 1, E and F). Protein profiling of 3 independent Pa-OMV preparations demonstrated highly consistent expression across batches (Figure 1G). Pa-STING CNPs remained stable in size for at least 2 weeks and exhibited no detectable cytotoxicity toward bone marrow-derived dendritic cells (BMDCs), airway epithelial cells, or human lung microvascular endothelial cells (Figure 1, H and I, and Supplemental Figure 1, D and E). These biophysical assessments demonstrate the precision, uniformity, and preliminary safety profile of the Pa-STING CNP vaccine candidate.

### Pa-STING nanoparticles activate antigen presenting cells.

**Figure 2:**
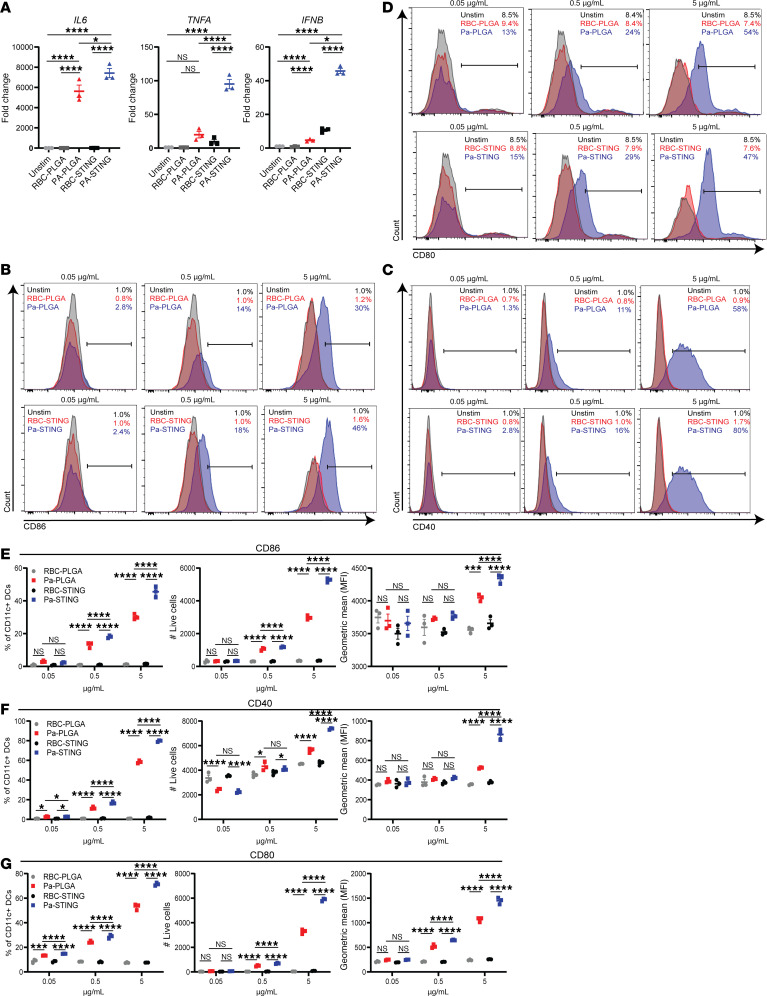
Pa-STING activates dendritic cells in a dose dependent manner. (**A**) Fold change in gene expression (qRT-PCR) of *IL6*, *TNFA*, and *IFNB* in BMDCs following stimulation with 5 μg/mL RBC-PLGA, Pa-PLGA, RBC-STING, or Pa-PLGA for 20 hours. Representative flow plots showing surface expression of (**B**) CD86, (**C**) CD40, and (**D**) CD80 from BMDCs stimulated with 0.05, 0.5, or 5 μg/mL RBC-PLGA, Pa-PLGA, RBC-STING, or Pa-STING. BMDCs were stimulated for 48 hours with indicated antigens, washed, stained, fixed, and analyzed by flow cytometry. Gates were drawn using single-stained, unstained, and fluorescence minus one (FMO) controls. BMDCs were gated live, CD11c^+^ and further gated on activation markers CD80, CD86, and CD40. (**E**–**G**) Quantification of **B**–**D** by percentage of CD11c^+^ DCs, total number of live cells, and geometric mean (gMFI), respectively. All panels representative of 3 independent experiments. (**A**, and **E**–**G**) analyzed by 2-way ANOVA with Šídák’s or Tukey’s multiple comparisons post test. Mean ± SEM. *P < 0.05, **P < 0.01, ***P < 0.001, ****P < 0.0001.

The cGAS-STING pathway is a critical innate immune sensor that induces inflammatory cytokines such as TNF-α and IL-6, as well as type 1 and 2 IFNs, in response to foreign DNA (48). To assess the contribution of the STING adjuvant to APC activation, we stimulated BMDCs with Pa-STING and RBC-STING nanoparticles and examined gene expression of STING pathway–dependent genes after 20 hours. Pa-PLGA and RBC-PLGA nanoparticles served as nonadjuvanted controls. Pa-STING CNPs robustly induced *IL6*, *TNFA*, and *IFNB* expression relative to nonadjuvanted Pa-PLGA controls (Figure 2A). In addition, Pa-STING stimulation significantly increased surface expression of DC activation markers CD86, CD40, and CD80 in a dose-dependent manner (Figure 2, B–D, Supplemental Figure 2). This enhanced activation was confirmed by 3 parameters: percentage of total CD11c^+^ DCs, total number of live DCs, and median fluorescence intensity (Figure 2, E–G). Collectively, these results demonstrate that the STING adjuvant confers a significant activation advantage to DCs.

**Figure 3:**
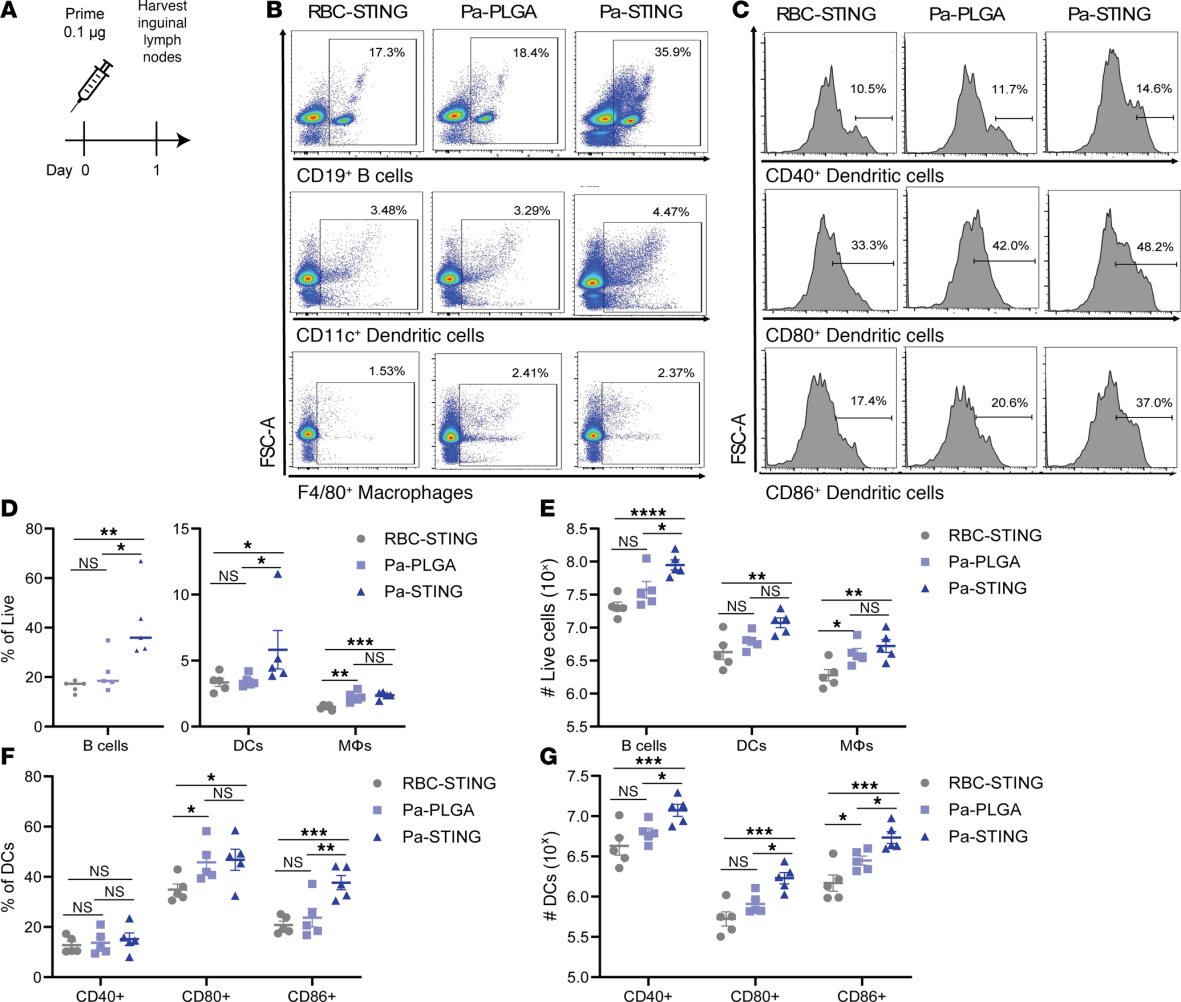
Pa-STING vaccination induces superior antigen presenting cell activation and recruitment to draining lymph nodes. (**A**) Mice were vaccinated with 0.1 μg RBC-STING, Pa-PLGA, or Pa-STING subcutaneously on the flank. 24 hours after vaccination, mice were humanely euthanized and inguinal lymph nodes were harvested, processed for single cell isolation, stained, and analyzed by flow cytometry. Gates were drawn using single-stained, unstained, and fluorescence minus one (FMO) controls. B cells were gated live, CD19^+^, macrophages (Mϕ) were gated live, F4/80^+^, dendritic cells (DCs) were gated live, CD11c^+^. DCs were further gated on activation markers CD80, CD86, and CD40. (**B**) Representative flow plots of B cells, DCs, and macrophages from vaccinated mice. (**C**) Representative flow plots of CD11c^+^ activated DCs. (**D** and **E**) Quantification of **B**. (**F** and **G**) Quantification of **C**. (**D**–**G**) analyzed by 2-way ANOVA with Šídák’s or Tukey’s multiple comparisons post test. *P < 0.05, **P < 0.01, ***P < 0.001, ****P < 0.0001.

To assess APC recruitment and activation in draining lymph nodes after vaccination, we isolated inguinal lymph nodes (ILNs) from mice 24 hours after subcutaneous vaccination in the flank with three CNPs: Pa-STING, Pa-PLGA (nonadjuvanted FDA approved polymer control), and RBC-STING (no OMV control) (Figure 3A). ILNs were processed for single-cell analysis, stained, and assessed by flow cytometry (Supplemental Figure 3). Pa-STING vaccination significantly increased the recruitment of B cells, DCs, and macrophages in ILNs, both by percentage and total number of live cells, compared with RBC-STING controls (Figure 3, B–E). Pa-STING also induced higher expression of activation markers CD40, CD80, and CD86 on CD11c^+^ DCs (Figure 3, C–G). Notably, Pa-STING vaccination elicited a superior immune response compared with nonadjuvanted Pa-PLGA, indicating that the STING adjuvant enhanced APC recruitment and activation. These findings are consistent with our in vitro data (Figure 2) and demonstrate that STING provides substantial adjuvant activity that enhances APC recruitment and activation.

**Figure 4:**
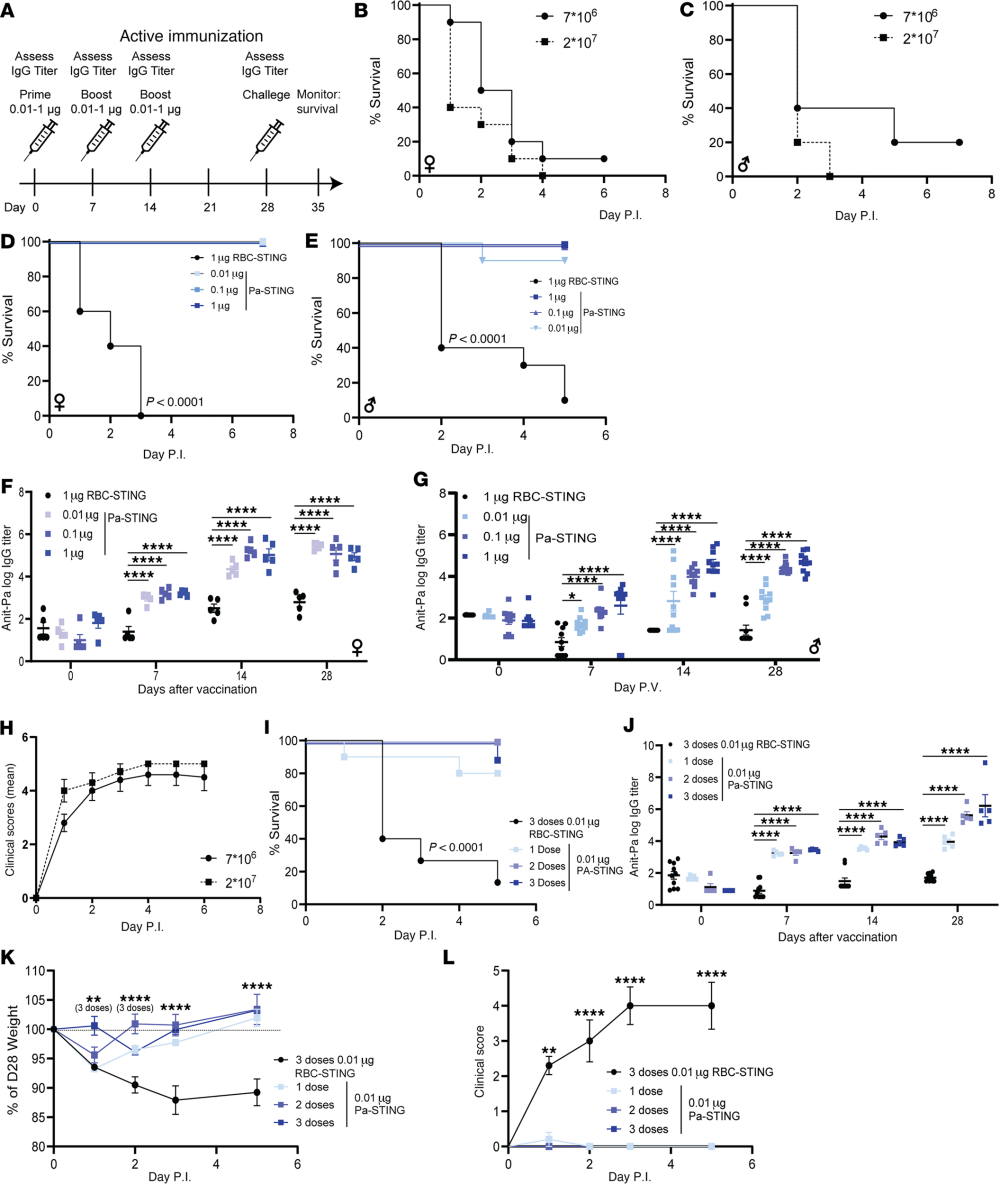
Pa-STING vaccination induces robust IgG responses and protects against lethal pneumonia. (**A**) Active immunization scheme with RBC-STING and Pa-STING, Mice were immunized subcutaneously with 0.01, 0.1, or 1 μg Pa-STING. Controls were immunized with the highest RBC-STING dose. Mortality curves in (**B**) female (♀) and (**C**) male (♂) nonimmunized mice identifying the effective lethal dose of PA14 pneumonia. Mice were infected with 0.7–2 × 10^7^ CFUs PA14 intratracheally and monitored for mortality for 7 days. *n* = 5/group, representative of 2 independent experiments. Mortality curves in (**D**) female (♀) and (**E**) male (♂) immunized mice infected with PA14. Mice were vaccinated with 0.01, 0.1, or 1 μg Pa-STING or 1 μg RBC-STING subcutaneously on days 0, 7, and 14. Mice were intratracheally infected with ~ 1 × 10^7^ CFUs PA14 on day 28. Morbidity and mortality were monitored twice daily for 7 days. *n* = 5–10/group. Representative of 2 independent experiments. (**F** and **G**) Anti-Pa IgG titers from (**D** and **E**), respectively. Titers were assessed by mandibular cheek bleeding and ELISAs on days 0, 7, 14, and 28. (**H**) Clinical daily scores for unvaccinated mice infected with 0.7–2 × 10^7^ CFUs PA14. Means ± SEM. *n* = 10/group, 2 independent experiments pooled. (**I**) Mortality curves in immunized mice infected with PA14. Mice were vaccinated with 1, 2, or 3 doses of 0.01 μg Pa-STING or 3 doses of 0.01 μg RBC-STING subcutaneously on days 0, 7, and 14. Mice were intratracheally infected with ~ 1 × 10^7^ CFUs PA14 on day 28. Mortality was monitored for 5 days. *n* = 10–15/group. Two independent experiments pooled. (**J**) Anti-Pa IgG titers from **I**. Each dot represents a mouse. *n* = 5–10 per group, representative of 2 independent experiments. (**K**) Percentage change in weight from day 28 and (**L**) clinical score in mice vaccinated and infected from **I**. (**D**, **E**, and **I**) Kaplan-Meier (Log-Rank) test. (**F**, **G**, and **J**–**L**) Mixed model 2-way ANOVA with Dunnet’s multiple comparisons post test. *P < 0.05, **P < 0.01, ***P < 0.001, ****P < 0.0001.

We next evaluated the safety and efficacy of Pa-STING vaccination in murine models. Mice were vaccinated subcutaneously with 3 doses of 1 μg RBC-STING or Pa-STING on days 0, 7, and 14 and monitored for safety and tolerability (Figure 4A). Both groups gained weight equally before and after vaccination (Supplemental Figure 4A). No differences were observed in serum creatinine or aspartate aminotransferase (AST) levels, which are biomarkers for renal and hepatic toxicity, respectively (Supplemental Figure 4, B and C). A complete blood count was performed on days –6, 1, 15, 28, and 42 to assess hematopoietic toxicity (Supplemental Figure 4, D–I, and Supplemental Table 1). A transient decrease in white blood count (WBC), neutrophil, and lymphocyte counts was observed in Pa-STING–vaccinated mice on day 15, but values remained within the typical range for B6 mice (Supplemental Figure 4, D–F) (49). The leukocyte counts quickly rebounded, and no consistent differences were observed between RBC- and Pa-STING–vaccinated mice (Supplemental Figure 4 and Supplemental Table 1). Comprehensive serum chemistry and hematology analyses on Day 29 revealed no changes in total WBC count, a slight increase in neutrophils, and a decrease in lymphocytes in Pa-STING–vaccinated mice; however, all values remained within the expected range for B6 mice (Supplemental Figure 5). Histopathological examination of the lung, thymus, kidney, adrenal glands, and sternum showed no significant abnormalities in either RBC-STING– or Pa-STING–vaccinated mice (Supplemental Figure 6). Multiple small inflammatory foci with infiltrating neutrophils in the liver, along with scattered individual cell necrosis in the spleen, were observed for both groups. Collectively, these results indicate that Pa-STING was well tolerated by mice and nontoxic to the hematopoietic system.

### Pa-STING vaccination protects against P. aeruginosa pneumonia morbidity and mortality.

To evaluate the efficacy of Pa-STING vaccination, mice received 3 subcutaneous doses of 0.01, 0.1, or 1 μg Pa-STING, with 1 μg RBC-STING as a control (Figure 4A). On day 28, mice were intratracheally challenged with approximately 1 × 10^7^ CFU of *P*. *aeruginosa* strain PA14, a dose that is lethal in approximately 90% of mice (Figure 4, B and C, and Supplemental Figure 7A). Remarkably, mice vaccinated with as little as 0.01 μg Pa-STING were fully protected from lethal pneumonia, whereas all RBC-STING–vaccinated controls succumbed within 3 days (Figure 4, D and E, and Supplemental Figure 7B). Assessment of serum antibody responses revealed dose-dependent anti-PA14 IgG titers on days 7 and 14 in both male and female mice (Figure 4, F and G). By day 28, IgG titers had equalized across all dose groups in female mice, while male mice continued to exhibit a dose-dependent response. This difference may reflect the use of uniform dosing across sexes without adjustment for the higher body weight typically observed in male mice.

To elucidate the impact of Pa-STING vaccination on infection-associated morbidity, we developed a clinical scoring system incorporating changes in body weight, temperature, mobility, and responsiveness to handling (Figure 4H, see Methods for additional details). Clinical scoring is particularly valuable in cases when interventions confer near-complete survival or when disease manifestations are mild, allowing for quantitative assessment of morbidity in the absence of mortality. To assess the minimal protective dose, mice were vaccinated with 1, 2, or 3 doses of 0.01 μg PA-STING, while RBC-STING served as controls (Figure 4I). Even a single dose of 0.01 μg Pa-STING provided 80% protection from lethal pneumonia. All 3 doses induced robust IgG responses, protected against infection-associated weight loss, and reduced clinical severity scores (Figure 4, I–L). Together, these results demonstrate that Pa-STING vaccination is highly effective at protecting mice from pneumonia, even with minimal antigen doses.

### Pa-STING vaccine protection is mediated by protective antibodies that promote bacterial clearance.

**Figure 5:**
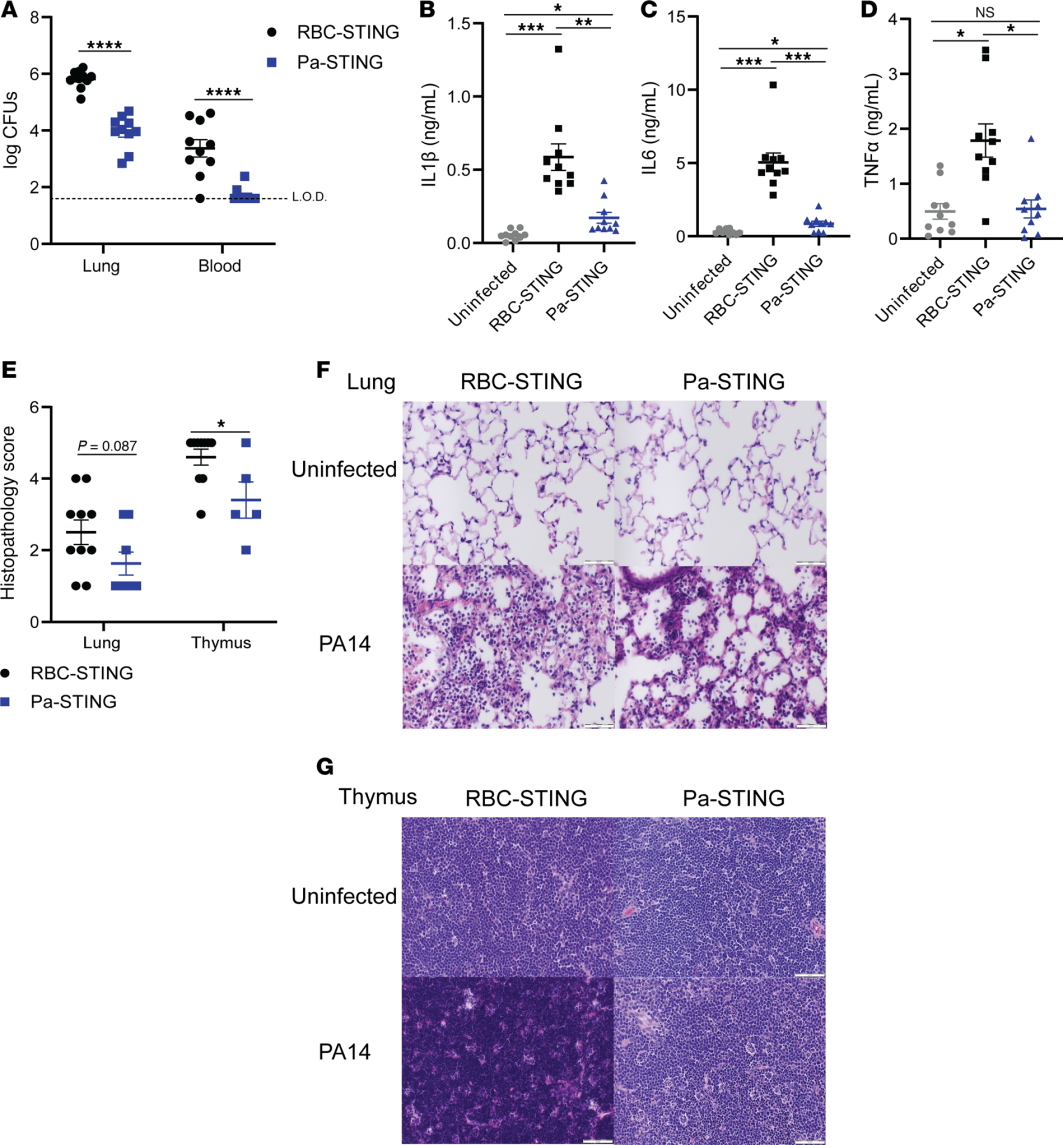
Pa-STING vaccination reduces bacterial load, inflammation, and pathology. Mice were immunized subcutaneously with 1 μg RBC-STING or Pa-STING. Uninfected mice were immunized with the 1 μg RBC-STING. Mice were infected with 0.7–2 × 10^7^ CFUs PA14 intratracheally on day 28. 20–24 hours after infection, mice were humanely euthanized. (**A**) Lungs and blood were collected, homogenized, serially diluted, and plated for enumeration. L.O.D., limit of detection. Bronchial alveolar lavage (BAL) fluid was collected and (**B**) IL-1β (**C**) IL-6 and (**D**) TNF-α levels were measured by ELISA. (**E**) Lungs were perfused with formalin, and thymus was fixed with formalin, embedded in paraffin blocks, sectioned, and stained for H&E. Scores were blindly assessed by a board-certified veterinary pathologist. Representative images of (**F**) lungs and (**G**) thymus from E. Scale bars: 50 μm. All panels were pooled from 2 independent experiments, *n* = 8–10/group. Each dot represents a mouse. (**A**–**D**) Mixed model 2-way ANOVA with Dunnet’s multiple comparisons post test. (**E**) Unpaired 2-tailed Student’s *t* test. *P < 0.05, **P < 0.01, ***P < 0.001, ****P < 0.0001.

To investigate the mechanism of protection conferred by Pa-STING vaccination, we assessed bacterial burdens 20 hours after infection. Mice vaccinated with 3 doses of 1 μg Pa-STING exhibited approximately a 2-log_10_-fold reduction in lung *P*. *aeruginosa* CFUs and nearly undetectable bacteremia relative to RBC-STING controls (Figure 5A). Pa-STING vaccination also significantly reduced levels of IL-1β, IL-6, and TNF-α in the bronchoalveolar lavage (BAL) fluid 20 hours after infection (Figure 5, B–G). Immune profiling of BAL fluid 20 hours after infection revealed a significantly higher percentage of eosinophils in RBC-STING–vaccinated mice, whereas no significant differences were observed in other immune cell populations (Supplemental Figures 8 and 9). Comprehensive hematology and serum chemistry analysis of both uninfected and infected mice showed mild leukopenia in both groups, primarily due to decreased lymphocyte counts (Supplemental Figure 5). Although some differences in cell populations and serum parameters were observed, most values remained within the normal reported range for C57BL/6 mice. These findings suggest that Pa-STING–mediated protection is not driven by shifts in cellular composition but instead by vaccine-elicited antibodies that enhance bacterial clearance and dampen inflammation.

**Figure 6:**
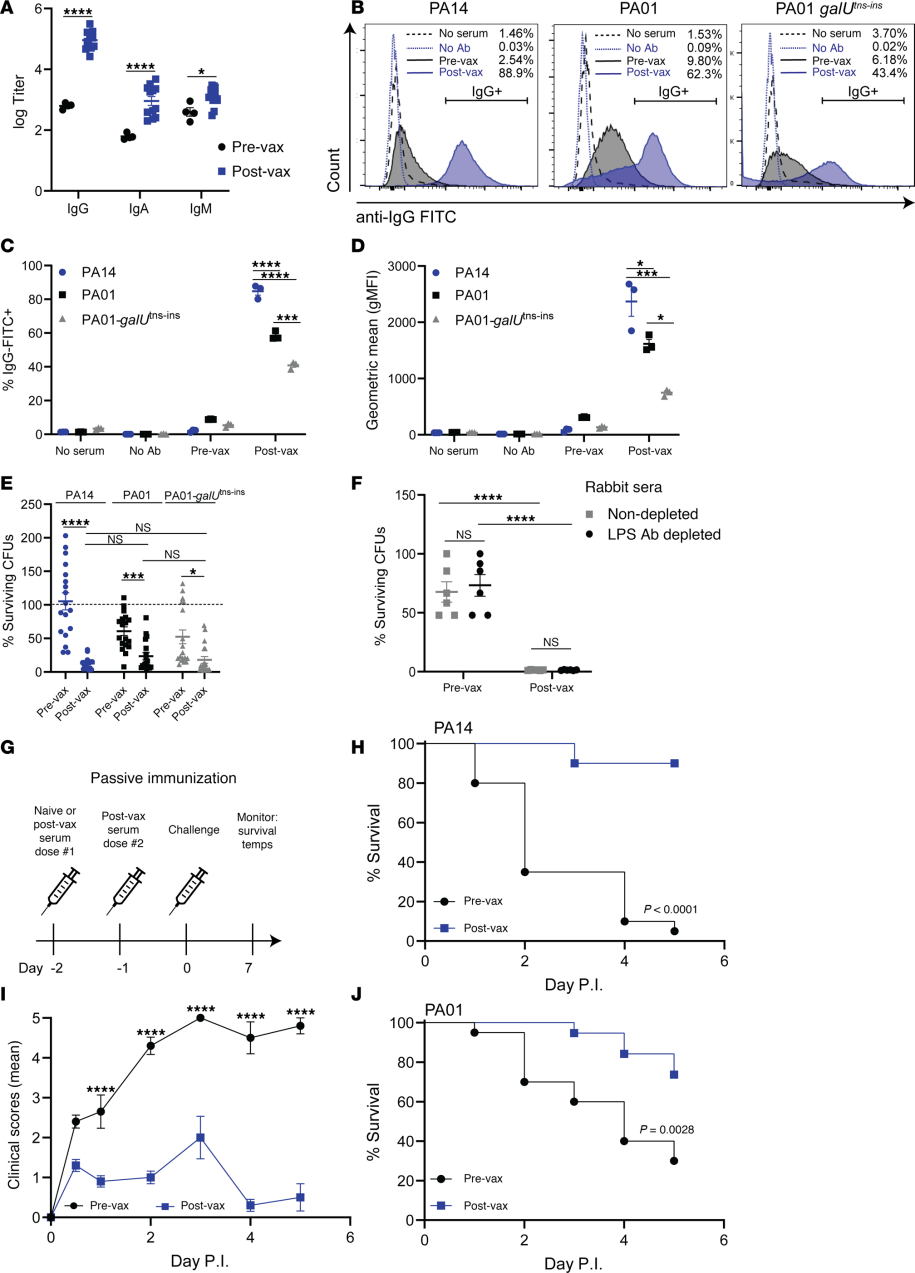
Pa-STING vaccination protection is antibody mediated and protects against infection with heterologous PA01. (**A**) IgG, IgA, and IgM antibody titers specific for PA14 from prevax and postvax serum obtained from two New Zealand white rabbits vaccinated with 4 successive doses of 0.25 mg Pa-STING subcutaneously with each dose 2 weeks apart (AbCore). Pooled means of technical replicates from 3–4 independent experiments ± SEM. (**B**) Representative histograms of anti-rabbit IgG FITC from prevax (black) and postvax (blue) serum bound to live PA14, PA01, and PA01-*galU^tns-ins^* measured by flow cytometry. Histograms are representative of technical replicates from 2–4 independent experiments. (**C**) % IgG-FITC positive of rabbit IgG and (**D**) geometric mean (median fluorescence intensity) of rabbit IgG from prevax and postvax serum bound to live PA14, PA01, and PA01*-galU^tns-ins^* measured by flow cytometry. Technical replicates from 2–4 independent experiments ± SEM. (**E**) Opsonophagocytic killing of PA14, PA01, and PA01-*galU^tns-ins^* by healthy human neutrophils incubated with prevax or postvax rabbit serum. Percentage surviving colony forming units (CFUs) relative to starting inputs is graphed. Pooled means of technical replicates from 3 independent experiments ± SEM. (**F**) Opsonophagocytic killing of PA14 by healthy human neutrophils incubated with non-depleted pre-vax or post-vax rabbit sera or sera depleted of LPS antibodies. (**G**) Passive immunization scheme with prevax or postvax rabbit serum. Mice received prevax or postvax intravenously 48 hours prior to infection PA14. (**H**) Survival curves from mice passively vaccinated with prevax or postvax serum 48 hours prior to intratracheal infection with ~ 1 × 10^7^ CFUs PA14. Mice were monitored twice daily for 5–7 days. *n* = 20/group, 2 independent experiments pooled. (**I**) Clinical scores (means ± SEM) from mice passively immunized with prevax or postvax serum and infected intratracheally with PA14. (**J**) Survival curves from mice passively vaccinated with prevax or postvax serum 48 hours prior to intratracheal infection with ~ 5 × 10^7^ CFUs PA01. Mice were monitored twice daily for 5–7 days. *n* = 20/group, 2 independent experiments pooled. (**A**) Two-way ANOVA with Dunnet’s multiple comparisons post test. (**C**–**E**) Mixed Model 2-way ANOVA with Tukey’s multiple comparisons post test. (**F**) Mixed Model 2-way ANOVA with uncorrected Fisher’s LSD. (**H** and **J**) Kaplan-Meier (Log-Rank) test. (**I**) Two-way ANOVA with Šídák’s multiple comparisons post test. *P < 0.05, **P < 0.01, ***P < 0.001, ****P < 0.0001.

To investigate the role of Pa-STING–induced antibodies in mediating protection, we vaccinated rabbits and collected pre- and postvaccination sera. Pa-STING significantly increased anti-Pa IgG titers (~2 log_10_-fold), with weaker IgA and IgM responses (Figure 6A). Postvaccination sera readily opsonized live PA14 as well as the heterologous *P*. *aeruginosa* strain PA01, indicating that Pa-STING generates cross-reactive antibodies (Figure 6, B–D). To assess the contribution of LPS to antibody recognition, we tested opsonization of a PA01-*galU^tns-ins^* mutant, which lacks a complete LPS core due to disruption of UDP-glucose pyrophosphorylase. Postvaccination sera opsonized PA01-*galU^tns-ins^* at approximately two-thirds the efficiency observed with WT PA01, suggesting that antibody recognition is partially LPS-dependent (Figure 6, B–D). When coincubated with live *P*. *aeruginosa* strains and healthy human neutrophils, postvaccination serum significantly enhanced neutrophil-mediated killing of PA14, PA01, and the LPS-deficient *galU* mutant (Figure 6E). To further assess LPS dependence, we depleted LPS-specific antibodies from both pre- and postvaccination sera. Notably, LPS-depleted postvaccination retained full opsonophagocytic activity (Figure 6F). Together, these findings indicate that Pa-STING vaccination elicits robust, functionally protective antibody responses capable of opsonizing diverse *P*. *aeruginosa* strains and promoting neutrophil-mediated bacterial clearance.

### Pa-STING vaccination protects against PA14 grown in artificial sputum media and CF clinical isolates.

Artificial sputum media (ASM) is a culture medium designed to mimic the physiologic environment of the CF lung. ASM contains amino acids, mucin, and extracellular DNA, and it induces altered secondary metabolite production in *P*. *aeruginosa* compared with conventional bacteriologic media (50). Importantly, Pa-STING postvaccination rabbit sera significantly enhanced opsonophagocytic killing of ASM-grown PA14 by healthy human neutrophils and conferred protection when passively transferred into mice subsequently infected with PA14 cultured in ASM (Supplemental Figure 10, A and F). Additionally, Pa-STING postvaccination sera improved neutrophil-mediated killing of 2 CF clinical isolates (Supplemental Figure 10G). Collectively, these findings demonstrate that antibodies elicited by Pa-STING vaccination promote neutrophil-dependent killing of *P*. *aeruginosa* strains grown in both bacteriologic and physiologically relevant media.

To test the protective capacity of these antibodies, we passively transferred pre- and postvax Pa-STING sera into mice and infected them intratracheally with PA14 2 days later (Figure 6G). Mice that received post-vax serum were protected from morbidity and mortality (Figure 6, H and I). Given the need for vaccines to protect against multiple strains, we also assessed cross protection by passively vaccinating mice with postvax Pa-STING sera and infecting with PA01, which significantly protected mice from lethal PA01 pneumonia (Figure 6J). These findings confirm that Pa-STING vaccination generates antibodies that bind and protect against both the homologous and a heterologous *P*. *aeruginosa* strain.

## Discussion

*P. aeruginosa* is a highly clinically relevant pathogen, notoriously difficult to treat due its inherent antibiotic resistance, ability to thrive in harsh environments, and the vulnerable patient populations it infects (51–53). Key mechanisms by which *P*. *aeruginosa* withstands host and therapeutic pressures include elevated spontaneous mutation rates, transition to a mucoid phenotype, modification or truncation of LPS O-antigen residues, antibiotic resistance via chromosomal mutations, and altered metabolic strategies (53). We have developed a *P*. *aeruginosa* OMV-based CNP vaccine candidate that protects mice from lethal pneumonia caused by the hypervirulent clinical isolate PA14 and a heterologous strain, PA01. Building on earlier work, where we stabilized an *A*. *baumannii* OMV membrane on an inert gold nanoparticle core (34), we initially applied this platform to develop a *P*. *aeruginosa* vaccine. However, Pa-NP vaccination conferred only partial protection against lethal infection and elicited variable antibody responses. These limitations led us to develop a next-generation, self-adjuvanted CNP vaccine incorporating a STING-activating core to enhance immunogenicity and protective efficacy.

Proper adjuvant selection is critical for vaccine design, as adjuvants can bias the immune response to either enhance or decrease protective immunity (54). We chose a STING-adjuvanted core, recently pioneered as a cancer metallotherapy and vaccine adjuvant (44, 45). The STING pathway, responsible for sensing foreign DNA, is a critical component of innate immunity and plays a key role in host defense against *P*. *aeruginosa* infections (43, 46, 47, 55). STING was selected for its ability to activate mucosal immunity, which is essential for defending against *P*. *aeruginosa* entry and infection (56–58). The fabricated Pa-STING CNPs were highly stable and precisely engineered for optimal uptake by APCs.

Pa-STING vaccination induced significantly higher recruitment of B cells, DCs, and macrophages in the ILNs 24 hours after vaccination compared with RBC-STING and nonadjuvanted Pa-PLGA controls. Moreover, Pa-STING significantly enhanced the activation of CD11c^+^ DCs, as shown by increased expression of CD80, CD86, and CD40. Comprehensive hematology, serum chemistry, and histopathological analysis revealed no vaccine-induced toxicity in the hematologic compartment or systemic organs. Remarkably, a single 0.01 μg dose of Pa-STING generated robust anti-*P*. *aeruginosa* IgG titers and protected mice from lethal pneumonia caused by the hypervirulent clinical isolate PA14. Passive immunization studies confirmed that protection was mediated by vaccine-induced antibodies, which also protected against a heterologous strain, PA01. Vaccine-induced antibodies promoted opsonophagocytic killing of PA14, PA01, and clinical CF isolates. Pa-STING vaccination additionally induced anti-*P*. *aeruginosa* IgA responses in rabbits, indicating successful engagement of mucosal immunity. These findings support the potential of OMV-stabilized CNP vaccines, further enhanced by adjuvanted cores, as a powerful platform for protection against *P*. *aeruginosa*.

Despite its considerable impact on global health, no vaccine against *P*. *aeruginosa* has reached the market. Preclinical and clinical candidates have largely focused on outer membrane proteins (OMPs) and lipopolysaccharide (LPS) as key antigens (56). However, the structural variability of the LPS O-antigen across approximately 20 serotypes complicates the development of a broadly protective vaccine (59–61). Side effects have derailed several otherwise immunogenic *P*. *aeruginosa* LPS-based vaccine candidates (62). One strategy to mitigate toxicity involves genetically detoxifying *P*. *aeruginosa* OMVs by deleting secreted toxins or modifying LPS structure (63). Other groups have engineered OMVs from heterologous bacteria to deliver *P*. *aeruginosa* antigens; however, neither approach addresses the challenge of generating a broadly cross-protective vaccine. OMV-based nanoengineered CNPs have the potential to overcome both of these challenges. The broad array of antigens in the OMVs can offer cross protection against heterologous strains from different serotypes, and complexing OMVs derived from multiple serotypes is easier and less costly than designing and producing multiple carbohydrate conjugates. Using OMV-based CNPs can also reduce the risk of LPS-induced toxicity due to the additional diversity of other lipids, proteins, and carbohydrates present on the membrane surface, and/or the potential to genetically detoxify LPS moieties in the producer *P*. *aeruginosa* strain before OMV collection. Given the high sensitivity of humans to LPS, its presence in our prototype Pa-STING vaccine represents a potential barrier to direct clinical translation. However, our studies using LPS-deficient mutants and antibody depletion indicate that antibody-mediated bacterial killing is not dependent on recognition of native LPS. These findings suggest that if future iterations of the vaccine require LPS detoxification or removal, protective efficacy may remain largely intact.

Beyond its high prevalence as an etiologic agent of hospital- and ventilator-associated pneumonia, *P*. *aeruginosa* is also a leading cause of lung failure and mortality in individuals with CF, one of the most common recessive genetic disorders associated with premature mortality (64). The introduction of the HEMT triple combo — elexacaftor/tezacaftor/ivacaftor (ETI) — which directly targets the defective cystic fibrosis transmembrane conductance regulator (CFTR) protein, has markedly improved clinical outcomes and quality of life in individuals with CF (42, 65, 66). In parallel with advances in screening and prevention, HEMT has significantly extended life expectancy for CF patients in the US since the 1990s (67). Despite these gains, *P*. *aeruginosa* colonization remains a persistent concern: as of 2021, approximately 50% of CF adults were colonized with *P*. *aeruginosa* (40, 41, 68, 69).

A vaccine targeting *P*. *aeruginosa* could offer critical protection for this highly vulnerable population. A limitation of our current study is that protective efficacy was evaluated only in young, immunocompetent mice, which do not fully recapitulate the clinical population at greatest risk. Although CFTR-deficient mouse models have been developed, they are more susceptible to infection, yet fail to exhibit the hallmark clinical manifestations of CF — viscous mucus obstructing the lungs, intestines, and other organs (70–72). To address this gap, we utilized artificial sputum media (ASM) which simulates the biochemical environment of CF airway secretions. *P*. *aeruginosa* grown in ASM display distinct metabolic features compared with growth in standard bacteriologic media (50).

Importantly, Pa-STING postvaccination rabbit sera retained opsonophagocytic activity against PA14 cultured in ASM and against clinical CF isolates. Moreover, passive immunization with Pa-STING sera conferred protection in mice challenged with ASM-grown PA14. These results suggest that growth in ASM does not substantially alter the antigenic composition of OMVs. Future studies will explore Pa-STING vaccination efficacy following pneumonia challenge in immunocompromised and aged murine models.

In summary, Pa-STING represents a highly effective CNP vaccine candidate capable of protecting against *P*. *aeruginosa* pneumonia and highlights the potential of adjuvanted cores to increase vaccine performance.

## Methods

### Sex as a biological variable

Our study included both male and female mice, as well as healthy human neutrophils isolated from consenting male and female donors. Comparable results were observed across sexes in both in vivo and in vitro experiments.

### Bacterial strains and cultures

PA14 was isolated from a burn wound patient at a hospital in Pennsylvania (73). PA01 was obtained from a wound in Melbourne, Australia in 1954 (74). PA01-*galU^tns-ins^* was a gift from Colin Manoil (UW Genome Sciences) (75). PA107 and PA108 were clinical isolates obtained from CF patients and provided by Ruth Siew (UC San Diego). Strains were grown in Luria Broth (LB, Sigma) at 37°C with aeration unless otherwise noted. Overnight stationary phase cultures were subcultured into fresh LB and grown at 37°C with aeration for ~ 3 hours until reaching mid-logarithmic phase (OD_600_ ~ 0.4–0.6) unless otherwise noted. Log-phase cultures were washed twice with PBS prior to use in experiments.

### Artificial Sputum Media (ASM)

ASM base media was prepared with 5 g/L gastric mucin (Pfaltz & Baue, #M32610), 4 g/L salmon sperm DNA (TCI America, #D35455G), 5.9 mg/L diethylene triamine pentaacetic acid, DTPA (TCI America, #D050425G), 5 g/L NaCl (Sigma), 2.2 g/L KCl (Sigma), 1.81 g/L Tris base (Sigma), 5 g/L casamino acids (Thermo Scientific, # AC612041000) (76). ASM base media was autoclaved, supplemented with 250 mg L-tryptophan (Sigma, prepared in 0.1M NaOH) and 5 mL 50% egg yolk emulsion (Neogen, #700004878), and stored at 4°C until use. Cultures grown in ASM were grown with aeration at 37°C identically to LB.

#### Pa-OMV derivation.

Single colonies of PA14 were inoculated into 1 L flasks of LB or 100 mL ASM and grown at 37°C with aeration for 16–20 hours. Stationary phase cultures were spun down at 10,000 RPM for 10 minutes in a Sorvall RC-6 refrigerated floor centrifuge. The supernatant was filtered through a 0.45 μm PES vacuum filter (Thermo Fisher, 167-0045) and concentrated ~ 100 x by tangential flow filtration (Repligen). Pa-OMVs were ultracentrifuged at 100,000*g* for 2 hours at 4°C in a Beckman Optima (XPN-90), resuspended in ultrapure water, and stored at –80°C.

### RBC membrane derivation

Mouse RBC membranes were derived according to established protocols (77, 78). Briefly, mouse RBCs (MSE00WBNH-0000637, BioIVT) were washed in 1 x EDTA/PBS 3 times, and the buffy coat layer was removed. Samples were aliquoted and stored at 80°C. For derivation, washed RBCs were thawed, resuspended in H_2_O and shaken vigorously for 5 min to lyse. Lysis continued at rest for an additional 10 minutes. Lysis was halted with 10 x PBS, and cells were spun down at >15,000*g* for 10 minutes at 4°C. Supernatant was aspirated, pellet was resuspended in H_2_O, and lysed for 5 minutes. Lysis was halted with 10 x PBS and cells were spun down at >15,000*g* for 5 minutes at 4°C. Lysis was repeated 1–2 x until the membrane pellet was clear with minimal residual RBC contamination. Membranes were pooled, lysed one additional time, resuspended in H_2_O, and protein concentration was measured by BCA (ThermoFisher). RBC membrane was resuspended at 5 mg/mL and stored at –80°C.

### Au-NP, PLGA, and STING synthesis

#### Gold (Au-NP) cores.

Citrate stabilized 30-nm AuNPs (NanoComposix, AUCN30) were mixed with RBC membranes or Pa-OMVs at a 1:1 weight ratio and bath sonicated for 2 minutes, as previously described (34). The mixture was then washed once with H_2_O by centrifugation at 5,000*g* for 15 minutes to remove any free OMVs or membrane.

#### PLGA polymers.

PLGA (poly DL-lactic-co-glycolic acid) polymers (50:50 PLGA, 0.67 dL/g; Lactel Absorbable Polymers) were created by rapid addition of PLGA at 10 mg/mL in acetone to equivalent volumes of water. Acetone was vacuum evaporated and resulting particles were resuspended at 10 mg/mL in dH_2_O.

#### STING polymers.

DSPE-H_11_ was synthesized by vortexing DSPE-maleimide (780201P-25 mg, Avanti Polar Lipids, 10 mg/mL in 95% EtOH) and CH_11_ (synthesized by Eton Biosciences, 100 uM in H_2_O) for 2 hours. Unbound peptides were dialyzed overnight at RT in 100% EtOH. Residual EtOH was vacuum evaporated, and DSPE-H_11_ was resuspended in DMSO (8 mg/mL). Manganese chloride tetrahydrate (Sigma, M87-500, 20 mg/mL in MeOH), c-di-AMP (Invivogen, tlr-nacda, 1 mg/mL in MeOH), and DSPE-H_11_ were mixed in methanol at a 50:7:12.5 volume ratio, sonicated in a bath sonicator, and vortexed overnight to produce STING particles. Particles were spun down at > 16,000*g* for 10 minutes, coated with Pa-OMVs or RBC membranes at 1:1 ratio by sonication in a bath sonicator for 2 minutes, and resuspended in 10% sucrose.

### Pa-STING characterization

Nanoparticle and OMV size, polydispersity index, and charge were assessed by dynamic light scattering (Malvern Zetasizer) with a refractive index of 2, dispersant as water. Stability was assessed by repeated DLS measurements over a 2-week period during storage at 4°C.

#### Cytotoxicity.

BMDC (DC2.4, Sigma SCC142), A549 (ATCC, CCL-185), and human lung microvascular endothelial cell viability (Sigma, 540-05A) were evaluated by incubating increasing concentrations of Pa-PLGA and Pa-STING in 96 well plates at 37°C, 5% CO_2_ for 48 hours. Viability was measured by PrestoBlue (ThermoFisher) or Cytotox 96 nonradioactive cytotoxicity assay (Promega) according to the manufacturer’s instructions. 1 mL stationary phase culture of LB or ASM grown PA14 was spun down, resuspended in PBS and NuPAGE LDS sample buffer (Invitrogen) and boiled for 10 minutes. OMV and lysate protein content was normalized by Pierce BCA Protein Assay Kit (ThermoFisher). 10 μg of OMV, lysate, or NP were run per lane and visualized by silver stain.

#### TEM.

5 μg/mL Pa-OMVS were laid on a Pelco EasiGLow-hydrophilized 400 mesh Formvar/Carbon-coated copper grid (0.1754-F, Electron Microscopy Sciences). Samples were negatively stained with 2% uranyl acetate (EM Sciences, USA). 5 μL of Pa-STING were laid on a nonhydrophilized 400 mesh Formvar/Carbon-coated copper grid (0.1754-F, Electron Microscopy Sciences) for 10 minutes at room temperature, followed by 3 30-second wash steps with distilled water and air dry. All samples were imaged under a JEOL JEM-1400Plus transmission electron microscope (Tokyo, Japan).

### DC activation

#### qRT-PCR.

24 hours after seeding in 24-well tissue culture treated plates, BMDCs (DC2.4, Sigma, SCC142) were stimulated with 5 μg/mL RBC-PLGA, RBC-STING, Pa-PLGA, Pa-STING, or left unstimulated. After 20–24 hours, the supernatant was aspirated, and RNA was isolated from cells with RNeasy Plus Mini Kit (Qiagen) with gDNA elimination according to the manufacturer’s protocols. 1 μg of RNA was reverse transcribed into cDNA (iScript gDNA Clear cDNA Synthesis Kit, BioRad). Quantitative real time PCR was run on a BioRad CFX96 with Perfecta SYBRMix (Quantabio). Fold change was determined using the 2ΔΔCt method with GAPDH as a housekeeping gene. IFNB For - TGGGTGGAATGAGACTATTGTTG, Rev - CTCCCACGTCAATCTTTCCTC; TNFA For - CCCTCACACTCAGATCATCTTCT, Rev - GCTACGACGTGGGCTACAG; IL6 For - TAGTCCTTCCTACCCCAATTTCC, Rev - TTGGTCCTTAGCCACTCCTTC; GAPDH For - AGGTCGGTGTGAACGGATTTG, Rev - TGTAGACCATGTAGTTGAGGTCA.

#### Flow Cytometry.

24 hours after seeding in 24-well tissue culture treated plates, BMDCs were stimulated with 0.05, 0.5, or 5 μg/mL RBC-PLGA, RBC-STING, Pa-PLGA, Pa-STING, or left unstimulated. 48 hours after stimulation, cells were harvested and stained for surface markers of DCs: CD11c (Invitrogen, 45-0114 82), and F4/80 (eBioscience, 25-4801-82) as well as the activation markers CD40 (BD, 562846), CD80 (BD, 561955), CD86 (BioLegend, 105011). Cells were also stained with Live/Dead Aqua (Invitrogen, L34957). Cells were run on a BD FACS Canto II and analysis was performed using FlowJo. Gates were drawn with single-stained, unstained, and fluorescence minus one (FMO) controls. Single color compensation was performed. A gating strategy is depicted in Supplemental Figure 2.

### Immunized rabbit sera

Two New Zealand White rabbits were immunized with 4 successive doses of 0.25 mg Pa-STING subcutaneously, at intervals of 2 weeks (AbCore). Rabbits were bled before (prevax) and after vaccination (postvax), serum was obtained, aliquoted and stored at –80°C.

### Murine and rabbit IgG, IgA, and IgM neutralization assays

#### ELISA.

ELISA, low binding Immulon 4 HBX 96-well plates (Thermo Fisher, 3855) were coated with ~ 1 × 10^8^ colony forming units (CFUs) of heat killed PA14 in sodium bicarbonate buffer (Sigma, C3041). Coated plates were incubated at 4°C overnight, washed with PBST three times, and blocked with 1 x reagent diluent #2 (RD#2, R&D Systems, DY995) for 2–4 hours at room temperature (RT). Rabbit or mouse sera was serially diluted in 1 x RD#2, added to blocked plates, and incubated overnight at 4°C. Plates were washed 3 x with PBST, incubated with goat anti-mouse IgG HRP (Southern Biotech, 1030-05), anti-rabbit IgG HRP (Southern Biotech, 4030-05), IgA (R&D Systems, HAF008), or IgM (Southern Biotech, 4020-05) in 1 x RD#2. Plates were incubated for 60–90 minutes at RT, washed 3 x with PBST, incubated with streptavidin in 1 x RD#2 (R&D Systems, DY998) for 30 minutes. Plates were washed 3 x with PBST, detected with TMB (BD, 555214). The reaction was halted with 2N (normal) H_2_SO_4_ (Sigma, 1.60313). Plates were read on a spectrophotometer at 450 nm. Antibody titer was determined using 4-parameter logistics curve fit (Prism).

#### Flow cytometry.

Flow cytometry, mid-log phase PA14, PA01, and PA01-*galE^tns-ins^* were blocked with 10% heat inactivated horse serum for 30 minutes 37°C, incubated with 2% prevax or postvax serum for 30 minutes at 37°C. Bacteria was washed with PBS, stained with goat anti-rabbit IgG AlexaFluor-488 (ThermoFisher, A-11008). Samples were washed, fixed with cytofix fixation buffer (BD, 554655), and run on a BD FACS Canto II. Gates were drawn using single-stained, prevax, and unstained controls.

### Opsonophagocytic killing assays

OPKs were performed as previously described with minor modifications (34). Healthy human neutrophils were isolated from consenting donors by venipuncture into sodium heparin vacutainers (BD, 367874). Blood was layered onto polymorph prep (Cosmo Bio, NC0863559), and neutrophils were isolated. RBCs were lysed with H_2_O, then neutrophils washed, counted, and resuspended in 1 × PBS at 1.1 × 10^6^ cells/mL. Mid-log phase bacterial cultures were washed 2 x with 1 x PBS and incubated with 10% prevax or postvax rabbit sera for 30 minutes at 37°C. Next, 2 × 10^5^ neutrophils were added to 96-well flat-bottomed untreated plates and opsonized bacteria were added at an MOI 0.1-1. Plates were spun at 1,000 RPM for 5 minutes and incubated at 37°C, 5% CO_2_. After 3 hours, wells were mixed thoroughly, lysed in H_2_O for 3 minutes, diluted in 1 × PBS, and plated on LA for enumeration.

#### LPS antibody depletion.

LPS antibody depletion was performed according to the method by Zollinger et al (79). Briefly, high-binding 96-well flat-bottomed plates were coated with 100 μg/mL purified *Pseudomonas* LPS (Sigma, SAB4200884) diluted in 1 × PBS+0.1% MgCl or buffer only. Plates were left uncovered at 37°C to evaporate overnight. The plate was washed 3 times with sterile Gey’s balanced salt solution+ 0.2% gelatin (GBSS, Sigma, G9779-500ML) for 90 minutes, shaking at 37°C. The plates were blocked overnight at 4°C with GBSS. One additional wash with GBSS for 90 minutes, shaking at 37°C, was conducted. Prevax and postvax rabbit sera were added to triplicate wells coated with either LPS (depleted) or PBS (nondepleted) and incubated shaking for 3–4 hours at 37°C. Sera was removed and immediately incubated with mid-log–phase bacterial cultures at 10% final sera concentration for opsonization as described above. The remaining OPK assay was performed without modifications.

### Murine studies

All animal studies were conducted in compliance with the Animal Welfare Act, applicable federal regulations, and the recommendations for care and use of laboratory animals instituted by the UCSD Institutional Animal Care and Use Committee (IACUC). All protocols were IACUC approved (#S00227M). Mice were housed in a specific pathogen free (SPF) on a 12-hour light/dark cycle. Mice received 2020X diet (Envigo), acidified water, and were housed in prebedded corn cob disposable cages (Innovive). Vivarium staff randomized mice into cages no less than 72 hours prior to experimentation, with up to 5 mice per cage.

### Hematology and safety studies

4–5 week-old C57BL/6 mice (Jackson Labs, 000664) were subcutaneously (SC) vaccinated with 1 μg RBC-STING or Pa-STING on days 0, 7, and 14. A comprehensive hematology panel was assessed on days –6, 1, 15, 28, and 42. Blood was obtained by mandibular cheek-bleeding from half of the cohort into lavender K_2_EDTA microtainers (Fisher, 02-669-33). A complete blood count was assessed on a Hemavet by the UCSD murine hematology and coagulation lab. Weight was assessed on days –6, 1, 15, 28, and 42. For renal and liver toxicity, blood was obtained by mandibular cheek bleeding into serum separator microtainers (BD, 02-675-185). Serum was isolated by centrifugation. Serum creatinine and aspartate aminotransferase (AST) was assessed with colorimetric serum assay kits (Cayman Chemical, NC0378620, 701640) according to the manufacturer’s instructions.

#### Comprehensive serum chemistry and hematology.

4–5 week-old male and female mice were vaccinated SC with 1 μg RBC-STING or Pa-STING on days 0, 7, and 14. Mice were infected with 0.5–2 × 10^7^ CFUs PA14 or left uninfected on Day 28. 20–24 hours postinfection, blood was collected by submandibular cheek bleeding and serum was isolated. Whole blood and serum were sent to IDEXX Technologies for analysis.

#### Histopathology.

Mice were vaccinated and treated as above. 20–24 hours postinfection, mice were humanely euthanized and the lungs were perfused with 10% formalin (Fisher, SF93-4). Lungs, liver, spleen, heart, thymus, adrenal glands, sternum, and kidneys were removed and fixed for 24 hours in 10% formalin, rinsed and stored to 70% EtOH prior to paraffin-block embedding, sectioning, and H&E staining by the UCSD Biorepository and Tissue Technology Shared Resources. Sections were blindly scored by a board-certified veterinary pathologist.

### Infection models

8–12 week-old C57BL/6 mice were infected with 0.5–2 × 10^7^ CFUs PA14 or 5 × 10^7^ CFUs PA01 intratracheally. Mice were anesthetized with 100 mg/kg ketamine and 10 mg/kg xylazine prior to infection and monitored on a heating pad until fully recovered from anesthesia. Mice were monitored twice daily for 5–7 days. Surface body temperatures were monitored by infrared thermometry every 12–24 hours. Clinical scores were determined by assessing changes to body weight, appearance and grooming, mobility, response to handling, body, and temperature. Scores of 5 were considered moribund and humanely euthanized with CO_2_ according to approved IACUC protocols.

#### CFUs, BAL.

20–24 hours p.i., mice were humanely euthanized. Bronchial alveolar lavage (BAL) was collected in 1 × PBS supplemented with 2 mM EDTA. Lungs were harvested, homogenized, and plated for enumeration. BAL was spun at 1,500 RPM for 10 minutes at 4°C to pellet infiltrating cells. IL1-β, TNF-α, and IL-6 were assessed in BAL supernatant by ELISA DuoSet according to the manufacturer’s instructions (R&D Systems). The BAL cell pellet was resuspended in 1 x PBS and counted by trypan blue exclusion. Cells were stained for the following cell markers CD3 (BD, 560591), CD11c, Siglec F (Biolegend, 155506), CD11b (Invitrogen, RM2828), Ly6G (Biolegend, 127614), MHCII (Biolegend, 107652), CD19 (BD557398), and Live/Dead Aqua for 30 minutes at room temperature. Cells were washed, fixed, and run on a BD FACS Canto II. Gates were drawn using single-stained, unstained, and FMO controls. Single color compensation was performed. A gating strategy is depicted in Supplemental Figure 8 (80).

#### Histopathology.

20–24 hours p.i., mice were humanely euthanized and the lungs were perfused with 10% formalin, fixed, embedded, mounted, stained, and scored as described above.

### Passive immunization

8–12 week-old C57BL/6 mice were passively immunized intravenously (retroorbitally) with 200 μL prevax or postvax rabbit sera (AbCore) with brief anesthesia by inhaled isoflurane. Mice were monitored until fully recovered from anesthesia. 48 or 72 hours after vaccination, mice were infected intratracheally with ~ 0.5–1 × 10^7^ CFUs PA14 or ~ 5 × 10^7^ CFUs PA01, as described above. Mice were monitored as above.

### STING vaccination

4–5 week-old B6 mice were vaccinated with 0.01, 0.1, or 1 μg RBC-STING or Pa-STING subcutaneously after brief anesthesia with inhaled isoflurane on days 0, 7, and 14. For dosing experiments, mice that received 1 or 2 doses received RBC-STING in lieu of Pa-STING. Weights and IgG antibody titers were assessed on days 0, 7, 14, and 28 by mandibular cheek bleeding and ELISA. Mice were infected on day 28 with ~ 1 × 10^7^ CFUs PA14 intratracheally as described above. Mice were monitored as above.

### ILN vaccination

6–7 week-old B6 mice were vaccinated with 0.1 μg RBC-STING, Pa-PLGA, or Pa-STING subcutaneously in the flank after brief anesthesia with isoflurane. 24 hours postvaccination, mice were humanely sacrificed, and inguinal lymph nodes were harvested. Lymph nodes were processed for single-cell isolation and stained for surface markers of antigen presenting cells CD19 (BD, 557398), CD11c, and F4/80 as well as the activation markers CD40, CD80, CD86, Cells were also stained with Live/Dead Aqua and Mouse FC Block. Cells were run on a BD FACS Canto II and analysis was performed using FlowJo. Gates were drawn with single-stained, unstained, and FMO controls. Single color compensation was performed. A gating strategy is depicted in Supplemental Figure 3.

### Statistics

Statistical analysis was performed using GraphPad Prism v10 based on the recommendation of a statistician. Comparisons of 2 groups were performed using 2-tailed Student’s *t* test or nonparametric test for normally or nonnormally distributed data, respectively. Three or more groups were compared with 1-way analysis of variance (ANOVA). Groups with multiple time points or pooled samples were analyzed with 2-way mixed-model ANOVA to control for batch effects. Matched samples were compared with 2-way repeated measures ANOVA or 2-tailed paired Student’s *t* tests. Survival analyses were conducted using log-rank (Mantel-Cox) tests for significance. *P* values < 0.05 were deemed statistically significant. FlowJo v10.2 was utilized for flow cytometry analysis. Gates were drawn using single-stained controls, FMOs, and unstained samples.

### Study approval

All animal studies were approved by UCSD IACUC, #S00227M prior to initiation of experimentation. All human studies were approved by UCSD IRB, #131002 prior to initiation of experimentation.

### Data availability

All data is located in the Supporting Data Value File.

## Author contributions

Co-first authorship: NK fabricated and analyzed the STING core, EB performed experiments, analyzed data, and wrote the manuscript. Conceptualization: EB and VN. Methodology: EB, NK, JZ, RHF, and VN. Investigation: EB, NK, TK, ATPN, JC, JO, IC, CHC, NC, SD, SMH, AS, JAZ, HB, DS, SY, SMH, and AM. Analysis: EB and NK. Writing: EB and VN. Editing: all authors. Supervision: RHF and VN. Funding: EB, RHF, and VN.

## Supplementary Material

Supplemental data

Unedited blot and gel images

Supporting data values

## Version 1 07/24/2025

In-Press Preview

## Version 2 09/09/2025

Electronic publication
