# Supplementary material for: STING-adjuvanted outer membrane vesicle nanoparticle vaccine against Pseudomonas aeruginosa
Source: JCI Insight. 2025 Jul 24;10(17):e188105. doi: 10.1172/jci.insight.188105 (PMC12487677; doi:10.1172/jci.insight.188105)
Supplement: Supplemental data [file jciinsight-10-188105-s236.pdf]

**Supplemental Information**

# A STING-Adjuvanted Outer Membrane Vesicle Nanoparticle Vaccine against *Pseudomonas aeruginosa*

Elisabet Bjånes[1,#], Nishta Krishnan[2,#], Truman Koh[1], Anh T.P. Ngo[1], Jason Cole[1], Joshua Olson[1], Ingrid Cornax[1], Chih-Ho Chen[1], Natalie Chavarria[1,†], Samira Dahesh[1,†], Shawn M. Hannah[1,†], Alexandra Stream[1,†], Jiaqi Amber Zhang[1,†], Hervé Besançon[1,†], Daniel Sun[1], Siri Yendluri[1], Sydney Morrill[1,], Jiarong Zhou[2], Animesh Mohapatra[2], Ronnie H. Fang[2], Victor Nizet[1,3]

[1]Division of Host-Microbe Systems and Therapeutics, Department of Pediatrics, University of California San Diego, La Jolla, California, USA

[2]Aiiso Yufeng Li Family Department of Chemical and Nano Engineering, University of California San Diego, La Jolla, California, USA

[3]Skaggs School of Pharmacy and Pharmaceutical Sciences, University of California San Diego, La Jolla, California, USA

**Contents**:

Supplemental Figure 1: Pa-NP vaccination with a gold core provides partial protection against lethal pneumonia

Supplemental Figure 2: Gating strategy for dendritic cell activation

Supplemental Figure 3: Gating Strategy for ILN Flow Cytometry

Supplemental Figure 4: Pa-STING vaccination does not induce toxicity in the hematology compartment

Supplemental Figure 5: Comprehensive serum chemistry and hematology from RBC-STING and Pa-STING vaccinated mice.

Supplemental Figure 6: Histopathology of RBC-STING and Pa-STING vaccinated mice

Supplemental Figure 7: No sex differences were observed in unvaccinated and vaccinated mice following intratracheal infection to PA14.

Supplemental Figure 8: Immune infiltrating cells in BAL in RBC-STING and Pa-STING vaccinated and PA14 infected mice.

Supplemental Figure 9: Gating Strategy for BAL Flow Cytometry

Supplemental Figure 10: Pa-STING vaccination provides protection against PA14 grown in artificial sputum media

Supplemental Table 1: Pa-STING vaccination does not induce toxicity in the hematology compartment.

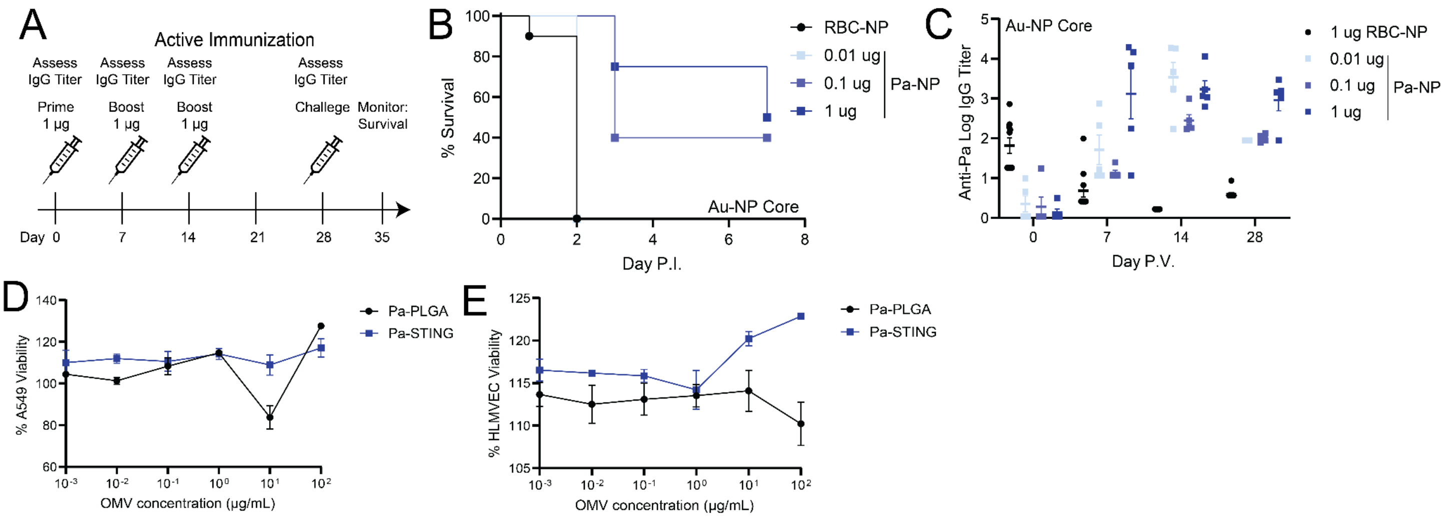


**Supplemental Figure 1: Pa-NP vaccination with a gold core provides partial protection against lethal pneumonia.** A) Active immunization scheme with RBC-NP and Pa-NP formulated with a gold core (Au-NP), Mice were immunized subcutaneously with 1 μg RBC-NP or Pa-NP. B) Mortality curves in mice vaccinated with 1 μg RBC-NP or Pa-NP and intratracheally infected with ~1 x $10^7$ CFUs PA14 pneumonia. Mice were monitored for mortality for seven days. C) Anti-Pa IgG titers from B). Titers were assessed by mandibular cheek bleeding and ELISAs on day 0, 7, 14, and 28. Means ± SEM. n=5/group, representative of one experiment. D) % A549 or E) % human microvascular endothelial cell (HMVEC) viability after incubation with Pa-STING or Pa-PLGA particles for 48 hours. Cells were incubated with increasing concentrations of Pa-STING or Pa-PLGA particles and viability was measured by CytoTox 96 non-radioactive cytotoxicity assay. Representative of three independent experiments.

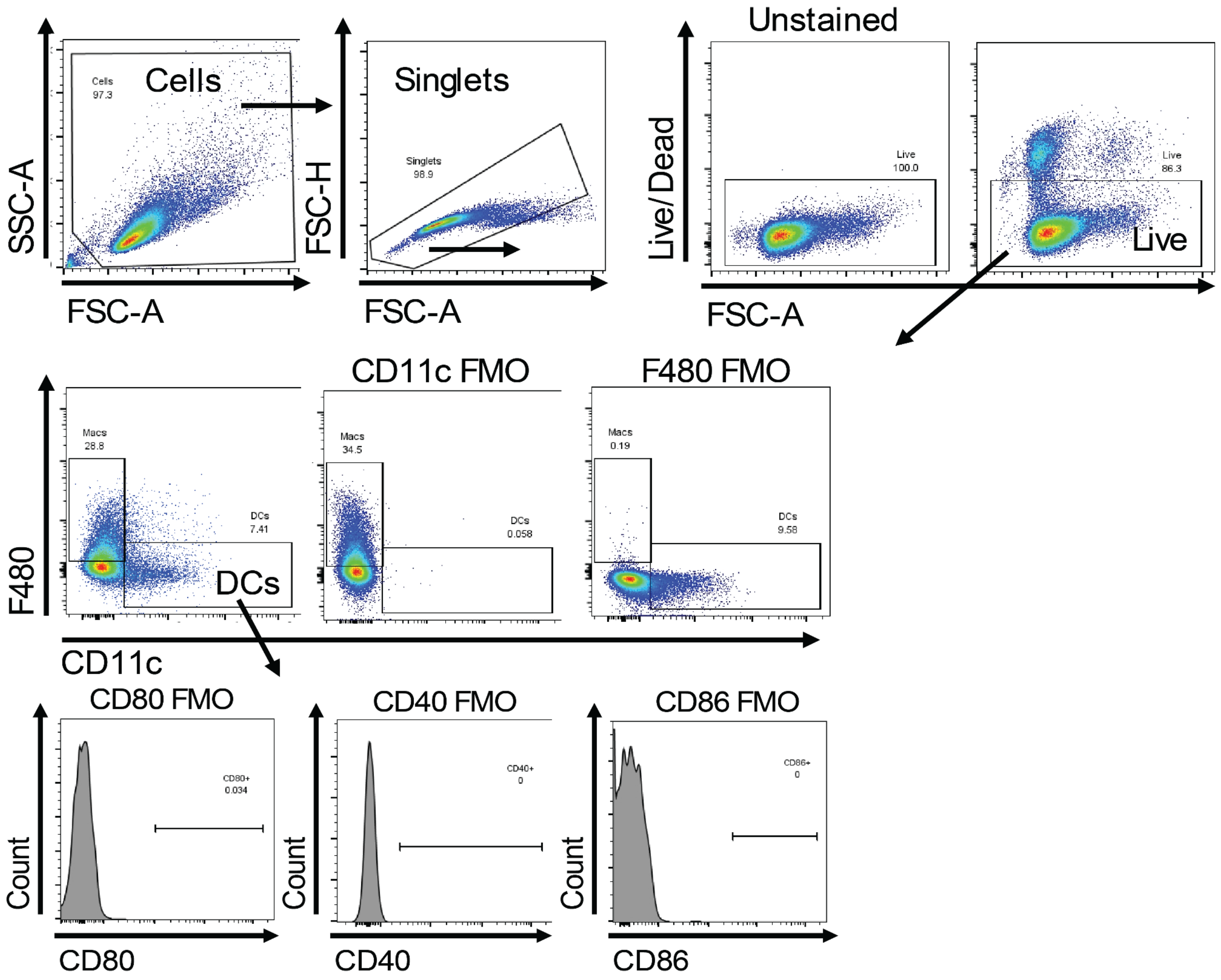


**Supplemental Figure 2: Gating strategy for dendritic cell activation.** For antigen presenting cell analysis, bone marrow derived dendritic cells were stimulated for 48 hours, harvested, stained and analyzed on a BD FACS Canto II. Cells were gated cells, singlets, live, CD11c$^{+}$/F4/80$^{-}$. Dendritic cells were further gated on CD86, CD40, and CD86. Gates were drawn with single-stained and unstained controls and with fluorescence minus ones.

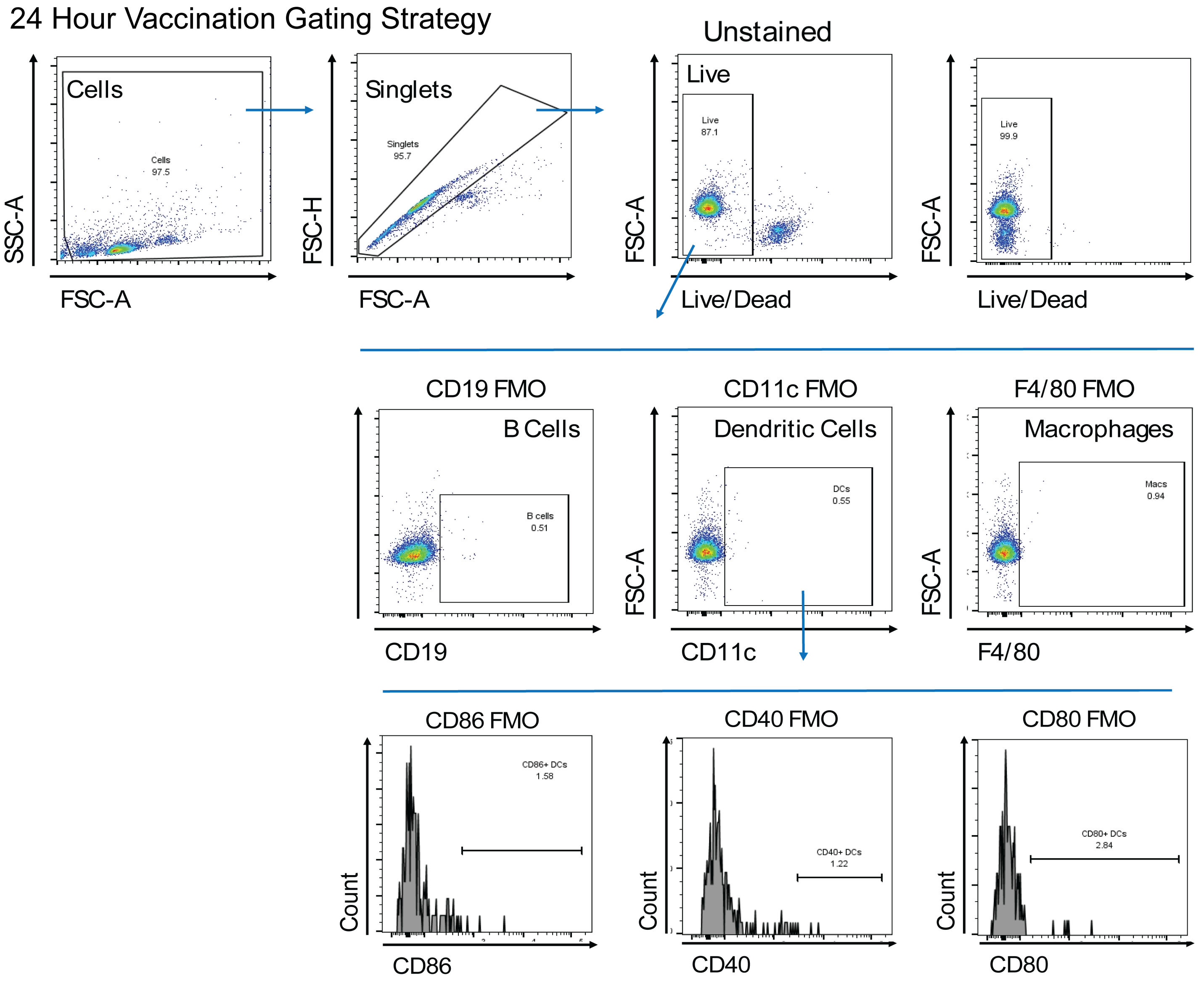


**Supplemental Figure 3: Gating Strategy for ILN Flow Cytometry.** For antigen presenting cell analysis, inguinal lymph nodes were processed for single cell isolation, stained and analyzed on a BD FACS Canto II. Cells were gated cells, singlets, live. B cells were gated CD19$^+$. Dendritic cells were gated CD11c$^+$. Macrophages were gated F4/80$^+$. Dendritic cells were further gated on CD86, CD40, and CD86. Gates were drawn with single-stained and unstained controls and fluorescence minus ones.

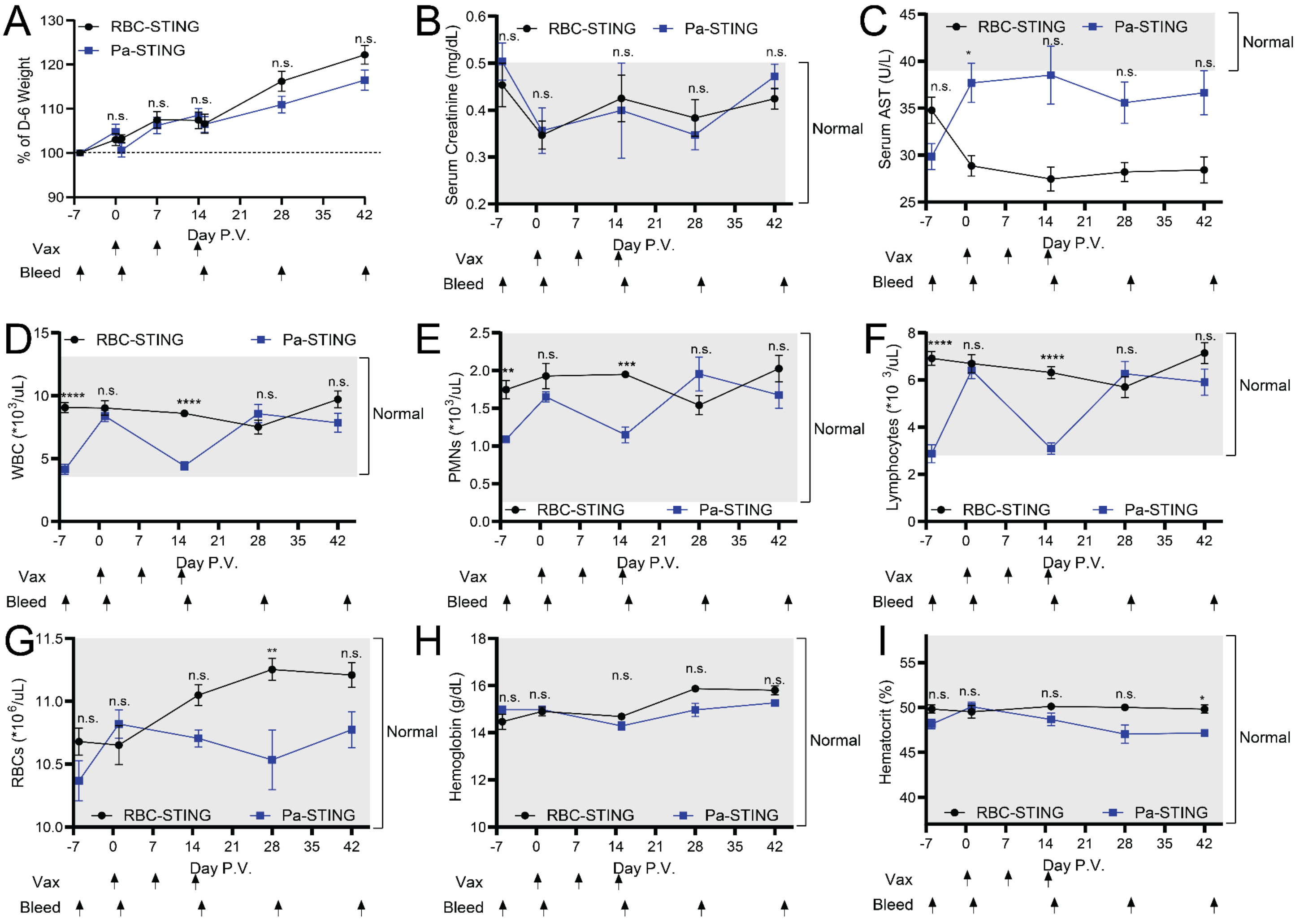


**Supplemental Figure 4: Pa-STING vaccination does not induce toxicity in the hematology compartment.** A) Percent weight gain during vaccination with 1 µg RBC-STING or Pa-STING. Mice were weighed on days -6, 0, 1, 7, 14, 15, 28, and 42. Serum B) creatinine and C) aspartate aminotransferase (AST) levels from mice vaccinated with 1 µg RBC-STING or PA-STING on days 0, 7, 14. Creatinine and AST levels were analyzed by Hemovet on days -6, 1, 15, 28, and 42. D-I) Hematology results from mice vaccinated with 1 µg RBC-STING or PA-STING on days 0, 7, 14. D) white blood count, E) polymorphonuclear cells (PMNs), F) lymphocytes, G) red blood cells (RBCs), H) hemoglobin, and I) hematocrit were analyzed by Hemovet on days -6, 1, 15, 28, and 42. Means ± SEM. n=5-10/group. (A-I) Two-way ANOVA with Sidak's multiple comparison's post-test. n.s. not significant. * $p < 0.05$, ** $p < 0.01$, *** $p < 0.001$, **** $p < 0.0001$. All data is listed in Supplemental Table 1 and Supplemental Data File.

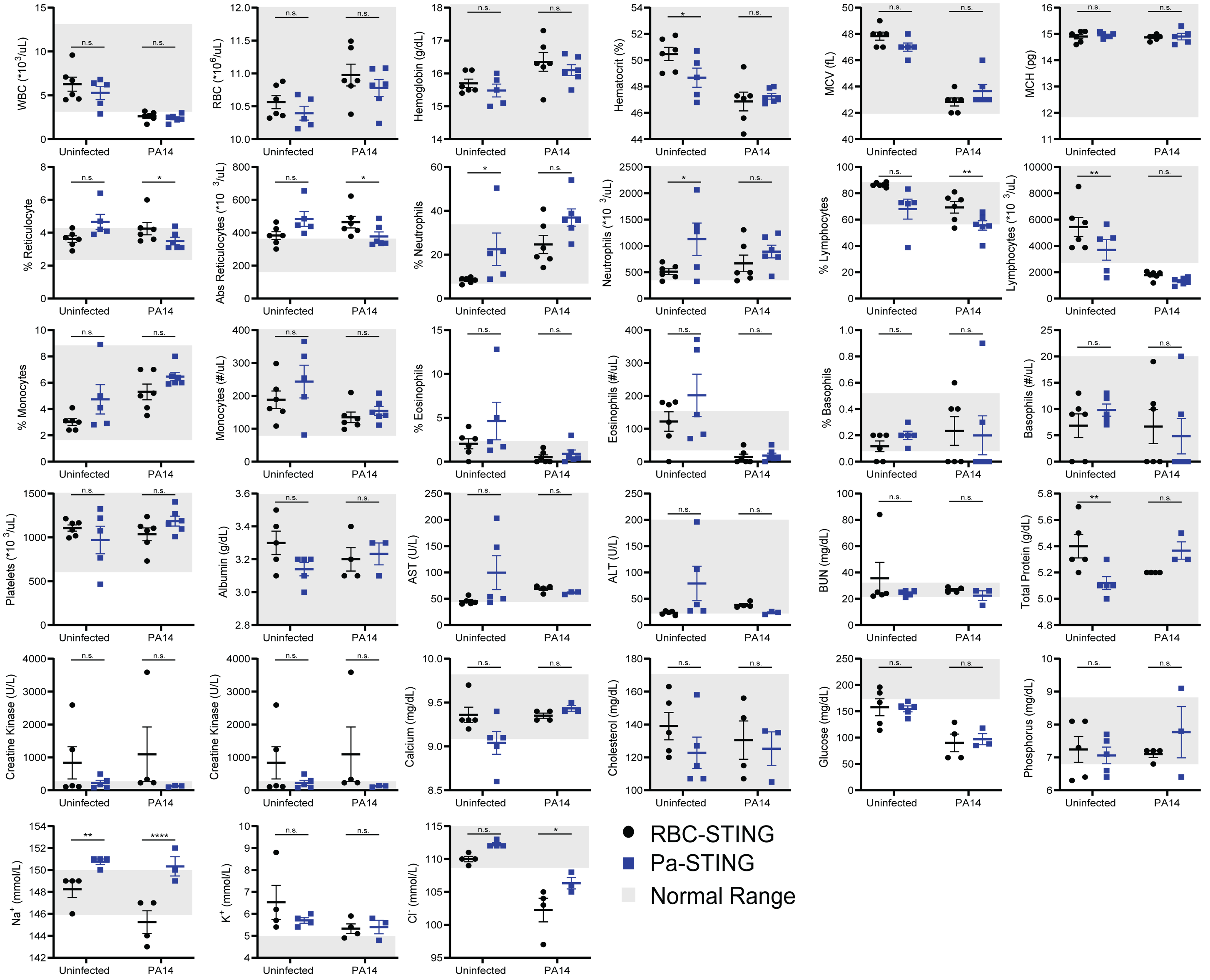


**Supplemental Figure 5: Comprehensive serum chemistry and hematology from RBC-STING and Pa-STING vaccinated mice.** Mice were vaccinated with 1 μg RBC-STING or Pa-STING on days 0, 7, and 14. Mice were infected with 0.5-1 x $10^7$ CFUs PA14 or left uninfected on day 28. On Day 29 blood was collected by submandibular cheek bleeding and serum was isolated. Whole blood and serum were sent for comprehensive serum chemistry and hematology by IDEXX Technologies. Some samples were pooled from two mice to reach the minimum volume necessary. The grey box indicates the normal expected range for B6 mice. Means ± SEM. n=5-6/group, two independent experiments pooled. Mixed model two-way ANOVA with uncorrected Fisher's LSD post-test. n.s. not significant. * $p < 0.05$, ** $p < 0.01$, *** $p < 0.001$, **** $p < 0.0001$. All data is listed in the Supplemental Data File.

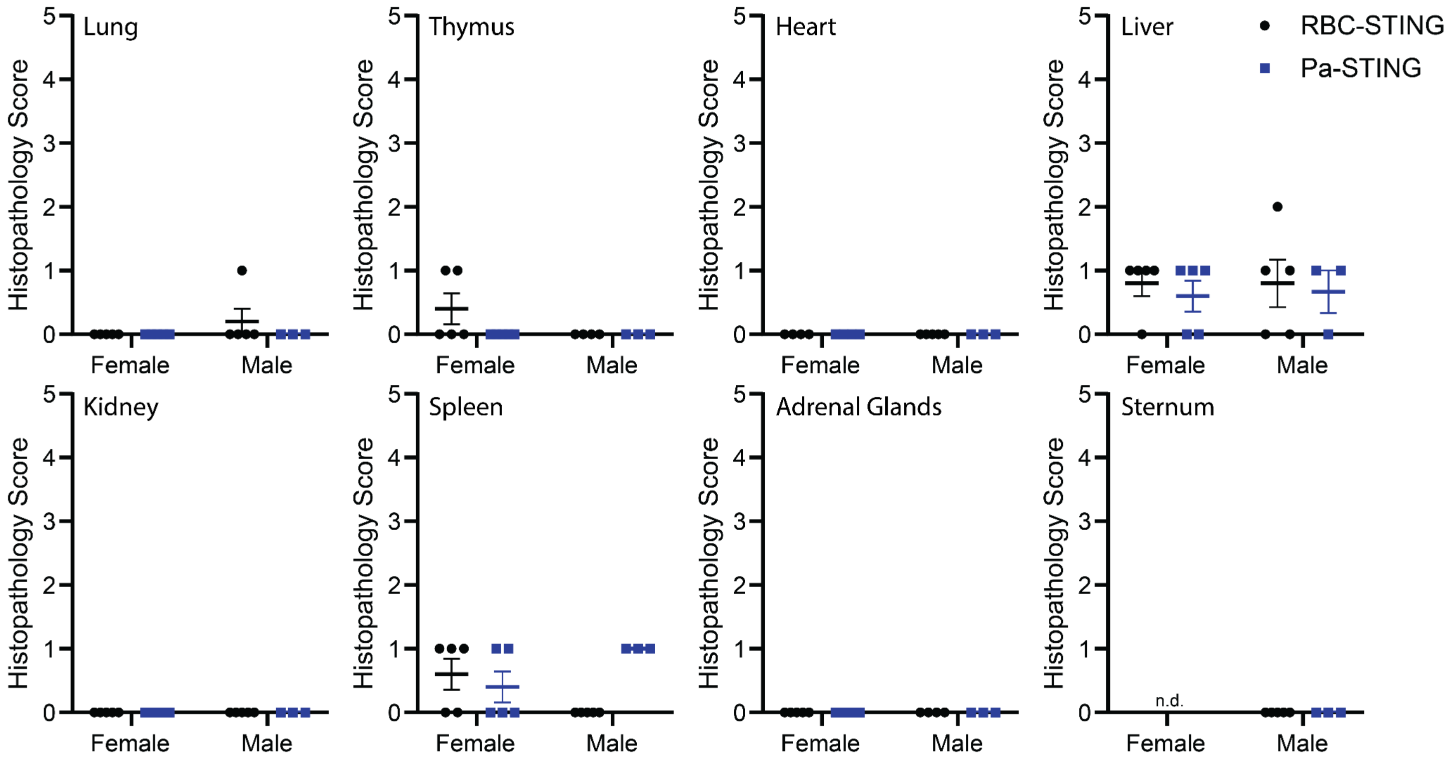


**Supplemental Figure 6: Histopathology of RBC-STING and Pa-STING vaccinated mice.** Mice were vaccinated with 1 µg RBC-STING or Pa-STING on days 0, 7, and 14. On Day 29 mice were humanely euthanized, the lungs were perfused with 10% formalin and removed along with the thymus, heart, liver, kidneys, spleen, adrenal glands, and sternum. Organs were fixed for 24 hours in 10% formalin, rinsed and stored to 70% EtOH prior to paraffin block embedding, sectioning, and H&E staining by the UCSD Biorepository and Tissue Technology Shared Resources. Sections were blindly scored by a board-certified veterinary pathologist. Two independent experiments pooled, n = 3-6/group.

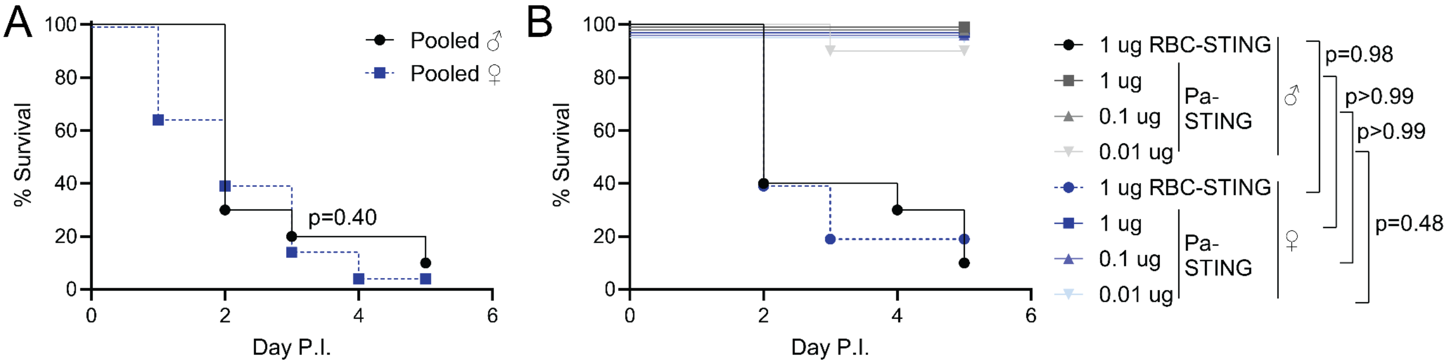


**Supplemental Figure 7: No sex differences were observed in unvaccinated and vaccinated mice following intratracheal infection to PA14.** A) Male and female survival data was pooled from **Figure 4B** and **4C** and analyzed using Kaplan-Meier (Log-rank). Median survival: male = 2.0 days, female = 2.0 days. B) Male and female survival data pooled from **Figure 4D** and **4E** and analyzed using Kaplan-Meier (Log-rank). Median survival: Male RBC-STING = 2.0 days, Female RBC-STING = 2.0 days. All other conditions had indeterminate median survival times.

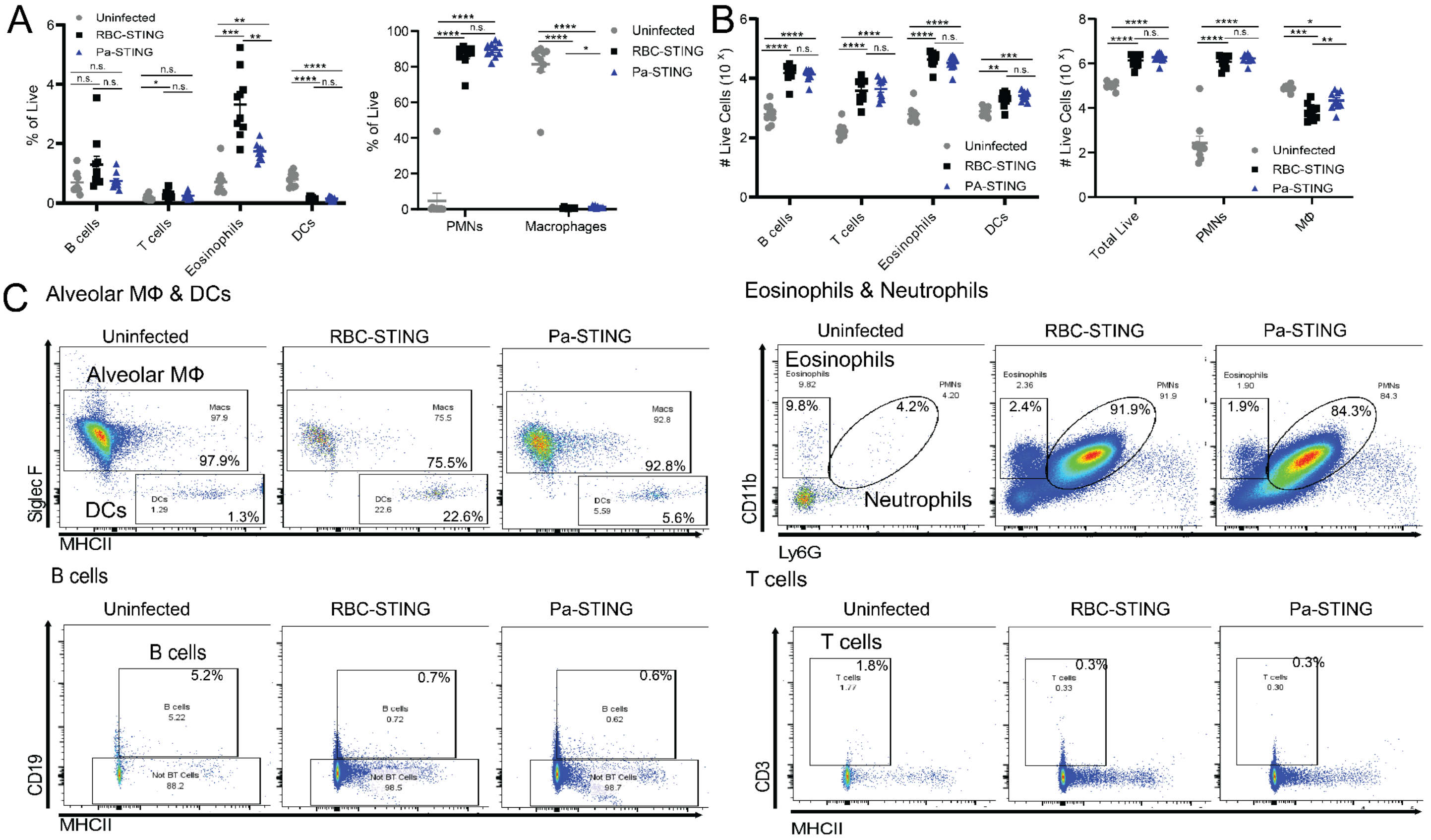


**Supplemental Figure 8: Immune infiltrating cells in BAL in RBC-STING and Pa-STING vaccinated and PA14 infected mice.** Mice were vaccinated with 1 µg RBC-STING or Pa-STING weekly on days 0, 7 & 14, and infected with ~0.5-1 x $10^7$ CFUs PA14 intratracheally on Day 28. RBC-STING vaccinated uninfected mice were used as a control. 20 hours p.i., mice were humanely euthanized and lung immune infiltrates were collected by BAL. Cells were stained, fixed and analyzed by flow cytometry. **A)** Quantification of cell populations as a percent of total live cells. **B)** Quantification of total number of live cells. A) & B) n = 10/group, two independent experiments pooled. Means ± SEM. **C)** Representative flow plots depicting cell populations in **A)** and **B)**. A gating strategy is depicted in **Supplemental Figure 9**. (A & B) Mixed model two-way ANOVA with Tukey's or Sidak's multiple comparisons post-test. n.s. not significant. * $p < 0.05$, ** $p < 0.01$, *** $p < 0.001$, **** $p < 0.0001$.

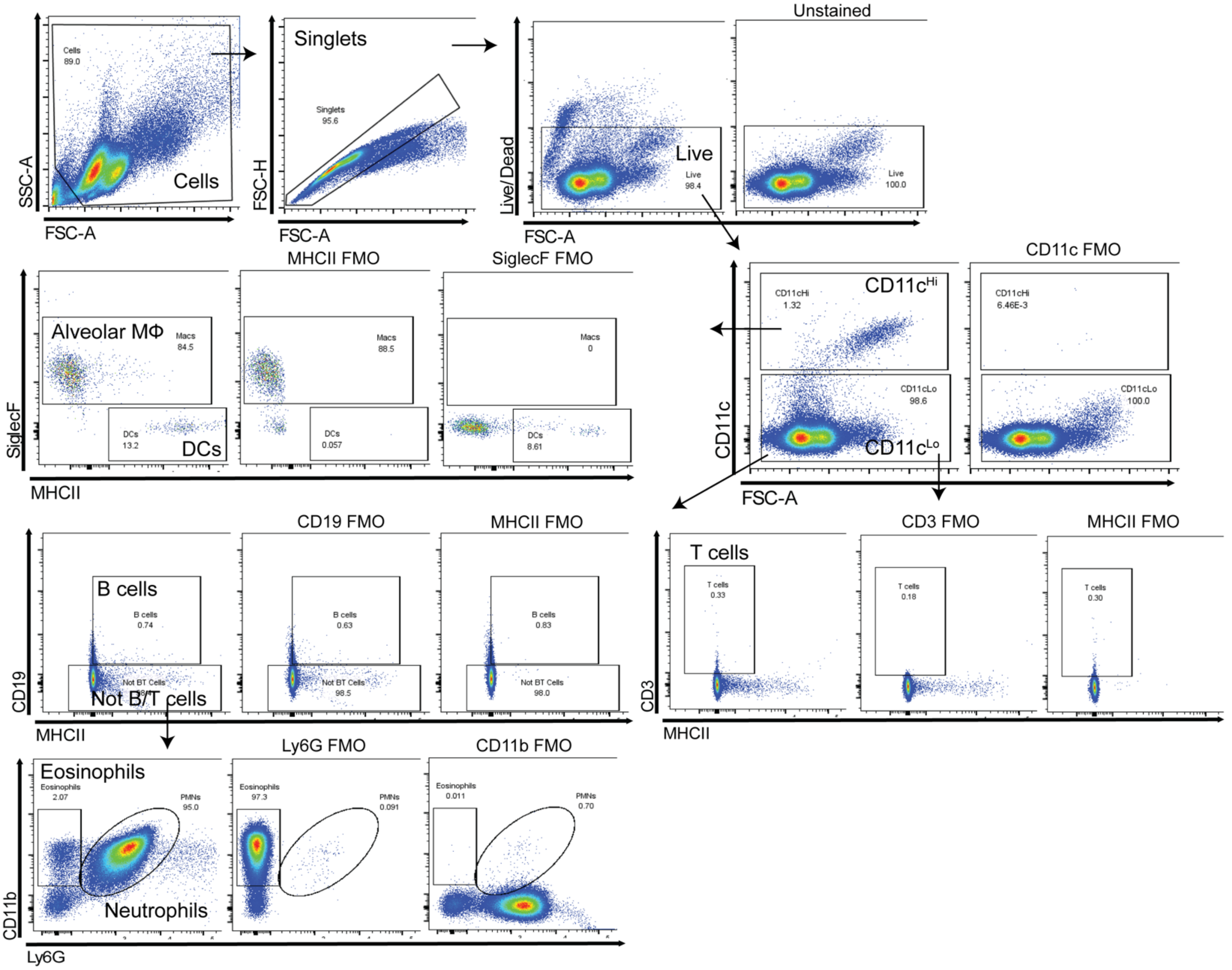


**Supplemental Figure 9: Gating Strategy for BAL Flow Cytometry.** For BAL infiltrating cell analysis, mice were humanely euthanized 24 hours post infection. BAL was collected, spun, stained and analyzed on a BD FACS Canto II. Gating strategy was based on the method by Van Hoecke *et al (1)*. Cells were gated cells, singlets, live, CD11c. Alveolar macrophages were gated $CD11c^{Hi}$, Siglec $F^{+}/MHCII^{-}$. Dendritic cells were gated $CD11c^{Hi}$, Siglec $F^{-}/MHCII^{+}$. T cells were gated $CD11c^{Lo}$, $CD3^{+}/MHCII^{-}$. B cells were gated $CD11c^{Lo}$, $CD19^{+}/MHCII^{+}$. Eosinophils were gated $CD11c^{Lo}$, $CD19^{-}$, $Ly6G^{-}/CD11b^{+}$. Neutrophils were gated $CD11c^{Lo}$, $CD19^{-}$, $Ly6G^{+}/CD11b^{+}$. Gates were drawn with single-stained and unstained controls and FMOs.

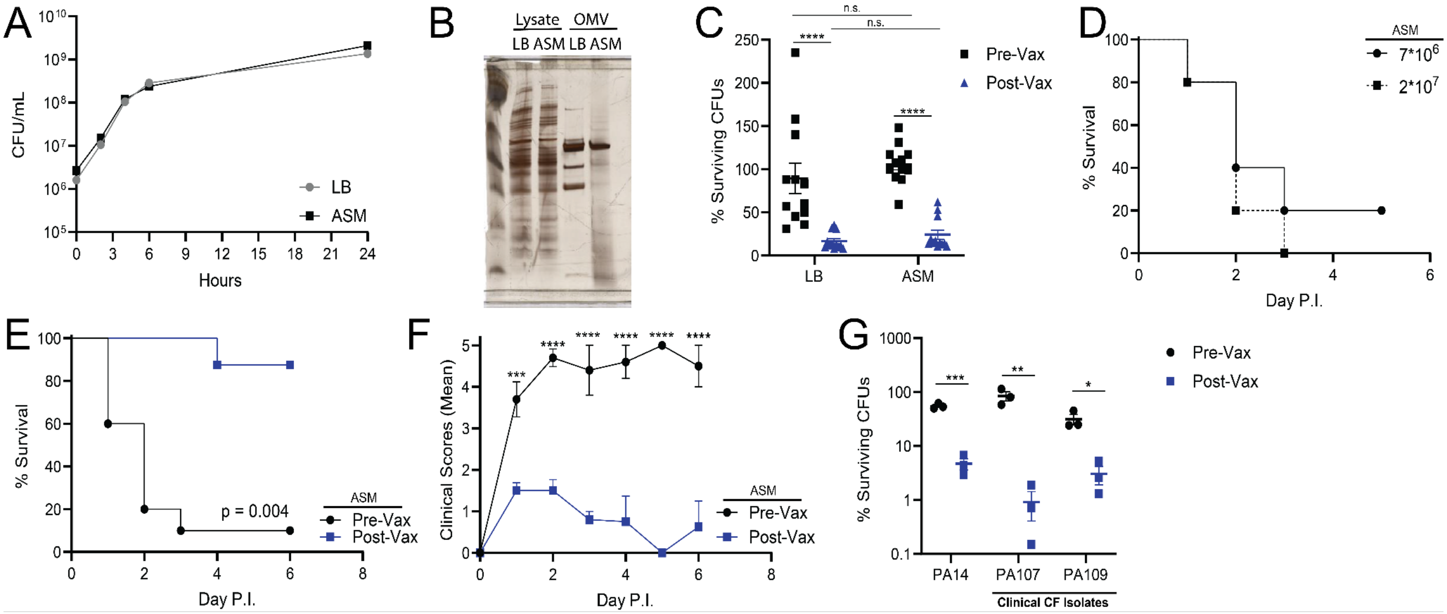


**Supplemental Figure 10: Pa-STING vaccination provides protection against PA14 grown in artificial sputum media.** A) Growth curves of PA14 grown in Luria broth (LB) or artificial sputum media (ASM). Representative of 2 independent experiments. B) Protein loading of LB vs ASM grown PA14 whole cell lysates and OMVs. ~10 µg of protein or lysate measured by BCA assay was loaded per lane and visualized with silver stain. C) Opsonophagocytic killing of PA14 grown in either LB or ASM by healthy human neutrophils incubated with pre-vax or post-vax rabbit serum. Percent surviving colony forming units (CFUs) relative to starting inputs is graphed. Pooled means of technical replicates from 2 independent experiments ± SEM. D) Survival curve of unvaccinated mice infected intratracheally with 0.7-2 x $10^7$ CFUs PA14 grown in ASM. E) Survival curves from mice passively vaccinated with pre-vax or post-vax serum 72 hours prior to intratracheal infection with ~0.5-1 x $10^7$ CFUs PA14 grown in ASM media. Mice were monitored twice daily for 5-7 days. n = 8-10/group, two independent experiments pooled. F) Clinical scores (means ± SEM) from mice passively immunized with pre-vax or post-vax serum and infected intratracheally with PA14 grown in ASM media. G) Opsonophagocytic killing of PA14 or clinical cystic fibrosis isolates PA107 and PA109 by healthy human neutrophils incubated with pre-vax or post-vax rabbit serum. Percent surviving colony forming units (CFUs) relative to starting inputs is graphed. C) Mixed model two-way ANOVA with Tukey's multiple comparisons post-test. E) Kaplan Meier (Log-Rank) test. F) Mixed model two-way ANOVA with Sidak's multiple comparisons post-test. G) Unpaired two-tailed Student's *t*-test. n.s. not significant. * $p < 0.05$, ** $p < 0.01$, *** $p < 0.001$, **** $p < 0.0001$.

**Supplemental Table 1: Pa-STING vaccination does not induce toxicity in the hematology compartment.**

| WBC (10^3/uL) | | | | | | WBC (%) | | | | | |
|---|---|---|---|---|---|---|---|---|---|---|---|
| | RBC | | PA | | | | RBC | | PA | | |
| **Day** | **Mean** | **± SEM** | **Mean** | **± SEM** | **p value** | **Day** | **Mean** | **± SEM** | **Mean** | **± SEM** | **p value** |
| **-6** | 9.056 | 0.4076 | 4.13 | 0.39563 | <0.0001 | **-6** | 9.056 | 0.4076 | 4.13 | 0.39563 | <0.0001 |
| **1** | 9.022 | 0.57995 | 8.376 | 0.45369 | 0.9225 | **1** | 9.022 | 0.57995 | 8.376 | 0.45369 | 0.9225 |
| **15** | 8.594 | 0.26322 | 4.42 | 0.33839 | 0.0002 | **15** | 8.594 | 0.26322 | 4.42 | 0.33839 | 0.0002 |
| **28** | 7.514 | 0.55218 | 8.566 | 0.72709 | 0.6261 | **28** | 7.514 | 0.55218 | 8.566 | 0.72709 | 0.6261 |
| **42** | 9.706 | 0.67084 | 7.844 | 0.75556 | 0.1151 | **42** | 9.706 | 0.67084 | 7.844 | 0.75556 | 0.1151 |

| PMNs (10^3/uL) | | | | | | PMNs (%) | | | | | |
|---|---|---|---|---|---|---|---|---|---|---|---|
| | RBC | | PA | | | | RBC | | PA | | |
| **Day** | **Mean** | **± SEM** | **Mean** | **± SEM** | **p value** | **Day** | **Mean** | **± SEM** | **Mean** | **± SEM** | **p value** |
| **-6** | 1.747 | 0.12195 | 1.089 | 0.03411 | 0.0084 | **-6** | 19.333 | 1.14094 | 27.41 | 1.9646 | 0.0524 |
| **1** | 1.928 | 0.16791 | 1.652 | 0.06472 | 0.595 | **1** | 21.291 | 0.94163 | 19.804 | 0.55751 | 0.7103 |
| **15** | 1.949 | 0.04534 | 1.148 | 0.10427 | 0.001 | **15** | 22.736 | 0.61595 | 26.064 | 1.53537 | 0.4018 |
| **28** | 1.54 | 0.12759 | 1.955 | 0.2262 | 0.184 | **28** | 20.512 | 0.76905 | 22.667 | 1.42202 | 0.7289 |
| **42** | 2.026 | 0.17707 | 1.675 | 0.17499 | 0.3405 | **42** | 20.854 | 0.88364 | 21.352 | 0.64677 | 0.9956 |

| Lymphocytes (10^3/uL) | | | | | | Lymphocytes (%) | | | | | |
|---|---|---|---|---|---|---|---|---|---|---|---|
| | RBC | | PA | | | | RBC | | PA | | |
| **Day** | **Mean** | **± SEM** | **Mean** | **± SEM** | **p value** | **Day** | **Mean** | **± SEM** | **Mean** | **± SEM** | **p value** |
| **-6** | 6.914 | 0.28951 | 2.869 | 0.38045 | <0.0001 | **-6** | 76.42 | 0.86052 | 68.193 | 2.35228 | 0.103 |
| **1** | 6.695 | 0.39156 | 6.408 | 0.36339 | 0.9919 | **1** | 74.355 | 1.00503 | 76.47 | 0.37278 | 0.4245 |
| **15** | 6.314 | 0.259 | 3.089 | 0.24714 | <0.0001 | **15** | 73.383 | 0.72448 | 69.798 | 1.98386 | 0.5558 |
| **28** | 5.7 | 0.4427 | 6.265 | 0.52641 | 0.864 | **28** | 75.784 | 0.78991 | 73.253 | 1.47895 | 0.6316 |
| **42** | 7.14 | 0.45044 | 5.909 | 0.5629 | 0.1736 | **42** | 73.766 | 1.34174 | 75.354 | 0.66602 | 0.8658 |

| Monocytes (10^3/uL) | | | | | | Monocytes (%) | | | | | |
|---|---|---|---|---|---|---|---|---|---|---|---|
| | RBC | | PA | | | | RBC | | PA | | |
| **Day** | **Mean** | **± SEM** | **Mean** | **± SEM** | **p value** | **Day** | **Mean** | **± SEM** | **Mean** | **± SEM** | **p value** |
| **-6** | 0.336 | 0.07231 | 0.138 | 0.00644 | 0.0013 | **-6** | 3.624 | 0.67849 | 3.415 | 0.38315 | 0.9997 |
| **1** | 0.345 | 0.03581 | 0.289 | 0.03367 | 0.7859 | **1** | 3.83 | 0.26487 | 3.412 | 0.22111 | 0.7801 |
| **15** | 0.278 | 0.03019 | 0.146 | 0.02561 | 0.0543 | **15** | 3.245 | 0.3721 | 3.309 | 0.47748 | >0.9999 |
| **28** | 0.252 | 0.01356 | 0.258 | 0.02663 | >0.9999 | **28** | 3.416 | 0.29992 | 3.043 | 0.2993 | 0.925 |
| **42** | 0.345 | 0.03138 | 0.223 | 0.03393 | 0.088 | **42** | 3.518 | 0.12141 | 2.788 | 0.25565 | 0.2001 |

| Eosinophils (10^3/uL) | | | | | | Eosinophils (%) | | | | | |
|---|---|---|---|---|---|---|---|---|---|---|---|
| | RBC | | PA | | | | RBC | | PA | | |
| **Day** | **Mean** | **± SEM** | **Mean** | **± SEM** | **p value** | **Day** | **Mean** | **± SEM** | **Mean** | **± SEM** | **p value** |
| **-6** | 0.041 | 0.01427 | 0.025 | 0.00447 | >0.9999 | **-6** | 0.449 | 0.14985 | 0.688 | 0.121 | 0.7648 |
| **1** | 0.4 | 0.26968 | 0.268 | 0.0142 | 0.8233 | **1** | 0.4 | 0.26968 | 0.268 | 0.0142 | 0.9948 |
| **15** | 0.039 | 0.0156 | 0.031 | 0.00678 | >0.9999 | **15** | 0.483 | 0.20024 | 0.656 | 0.09921 | 0.9578 |
| **28** | 0.018 | 0.00339 | 0.072 | 0.02601 | 0.9958 | **28** | 0.25 | 0.05753 | 0.828 | 0.29223 | 0.4698 |
| **42** | 0.151 | 0.05073 | 0.03 | 0.00474 | 0.8695 | **42** | 1.459 | 0.41514 | 0.426 | 0.08549 | 0.2911 |

| Basophils (10^3/uL) | | | | | | Basophils (%) | | | | | |
|---|---|---|---|---|---|---|---|---|---|---|---|
| | RBC | | PA | | | | RBC | | PA | | |
| **Day** | **Mean** | **± SEM** | **Mean** | **± SEM** | **p value** | **Day** | **Mean** | **± SEM** | **Mean** | **± SEM** | **p value** |
| **-6** | 0.015 | 0.00652 | 0.01 | 0.00354 | 0.9958 | **-6** | 0.172 | 0.06486 | 0.293 | 0.07957 | 0.7979 |
| **1** | 0.012 | 0.00718 | 0.004 | 0.00187 | 0.9656 | **1** | 0.125 | 0.06762 | 0.043 | 0.00982 | 0.8244 |
| **15** | 0.012 | 0.00957 | 0.007 | 0.00122 | 0.9958 | **15** | 0.156 | 0.11646 | 0.174 | 0.03562 | >0.9999 |
| **28** | 0.001 | 0.001 | 0.018 | 0.00735 | 0.5483 | **28** | 0.036 | 0.01111 | 0.21 | 0.08404 | 0.4321 |
| **42** | 0.043 | 0.02004 | 0.004 | 0.001 | 0.0078 | **42** | 0.401 | 0.169 | 0.078 | 0.01158 | 0.4974 |

| RBCs (10^6/uL) | | | | | | Platelets (10^3/uL) | | | | | |
|---|---|---|---|---|---|---|---|---|---|---|---|
| | RBC | | PA | | | | RBC | | PA | | |
| **Day** | **Mean** | **± SEM** | **Mean** | **± SEM** | **p value** | **Day** | **Mean** | **± SEM** | **Mean** | **± SEM** | **p value** |
| **-6** | 10.679 | 0.10612 | 10.368 | 0.16004 | 0.4325 | **-6** | 608.1 | 16.9192 | 420.5 | 32.9981 | 0.0117 |
| **1** | 10.652 | 0.1544 | 10.819 | 0.11111 | 0.9094 | **1** | 643.9 | 16.1736 | 635.2 | 21.9696 | 0.9992 |
| **15** | 11.05 | 0.08204 | 10.706 | 0.0677 | 0.3255 | **15** | 688.1 | 21.7373 | 585.6 | 63.7465 | 0.6502 |
| **28** | 11.253 | 0.08773 | 10.534 | 0.23711 | 0.0023 | **28** | 768.2 | 15.9833 | 727.3 | 67.833 | 0.9878 |
| **42** | 11.209 | 0.09861 | 10.774 | 0.14135 | 0.1252 | **42** | 705.7 | 24.6939 | 669.1 | 52.768 | 0.9824 |

| Hemoglobin (g/dL) | | | | | | Hematocrit (%) | | | | | |
|---|---|---|---|---|---|---|---|---|---|---|---|
| | RBC | | PA | | | | RBC | | PA | | |
| **Day** | **Mean** | **± SEM** | **Mean** | **± SEM** | **p value** | **Day** | **Mean** | **± SEM** | **Mean** | **± SEM** | **p value** |
| **-6** | 14.47 | 0.32117 | 14.98 | 0.14883 | 0.6779 | **-6** | 49.81 | 0.47021 | 48.16 | 0.54139 | 0.2303 |

| | | | | | | | | | | | |
|---|---|---|---|---|---|---|---|---|---|---|---|
| **1** | 14.9 | 0.18908 | 14.98 | 0.09028 | 0.9982 | **1** | 49.52 | 0.70331 | 50.08 | 0.53024 | 0.9803 |
| **15** | 14.69 | 0.06782 | 14.29 | 0.17847 | 0.3721 | **15** | 50.11 | 0.36242 | 48.66 | 0.70735 | 0.4668 |
| **28** | 15.87 | 0.06819 | 14.97 | 0.27776 | 0.1395 | **28** | 50.01 | 0.40847 | 47.04 | 1.00603 | 0.1791 |
| **42** | 15.8 | 0.19685 | 15.27 | 0.13657 | 0.2736 | 42 | 49.81 | 0.457 | 47.16 | 0.38968 | 0.0119 |

| **MCV (fL)** | | | | | | **MCH (Pg)** | | | | | |
|---|---|---|---|---|---|---|---|---|---|---|---|
| | **RBC** | | **PA** | | | | **RBC** | | **PA** | | |
| **Day** | **Mean** | **± SEM** | **Mean** | **± SEM** | **p value** | **Day** | **Mean** | **± SEM** | **Mean** | **± SEM** | **p value** |
| **-6** | 46.65 | 0.39718 | 46.46 | 0.24052 | 0.9974 | -6 | 13.57 | 0.36042 | 14.45 | 0.12748 | 0.3034 |
| **1** | 46.48 | 0.27046 | 46.3 | 0.08367 | 0.9824 | 1 | 13.99 | 0.14612 | 13.84 | 0.14089 | 0.9624 |
| **15** | 45.34 | 0.21413 | 45.44 | 0.54369 | >0.9999 | 15 | 13.31 | 0.09798 | 13.35 | 0.16733 | >0.9999 |
| **28** | 44.44 | 0.16233 | 44.67 | 0.51904 | 0.9972 | 28 | 14.1 | 0.07906 | 14.22 | 0.1437 | 0.9658 |
| **42** | 44.45 | 0.23184 | 43.81 | 0.42024 | 0.7277 | 42 | 14.1 | 0.16508 | 14.19 | 0.2176 | 0.999 |

| **Serum Creatinine (mg/dL)** | | | | | | **Serum AST (U/L)** | | | | | |
|---|---|---|---|---|---|---|---|---|---|---|---|
| | **RBC** | | **PA** | | | | **RBC** | | **PA** | | |
| **Day** | **Mean** | **SEM** | **Mean** | **SEM** | **p value** | **Day** | **Mean** | **SEM** | **Mean** | **SEM** | **p value** |
| **-6** | 0.45357 | 0.04669 | 0.50357 | 0.03929 | 0.9434 | -6 | 34.7672 | 1.39227 | 29.8479 | 1.4087 | 0.1756 |
| **1** | 0.34643 | 0.02963 | 0.35625 | 0.04877 | >0.9999 | 1 | 28.8573 | 1.10811 | 37.691 | 2.08453 | 0.0458 |
| **15** | 0.425 | 0.05018 | 0.39911 | 0.10125 | 0.9998 | 15 | 27.4607 | 1.28956 | 38.5227 | 3.09373 | 0.0934 |
| **28** | 0.38304 | 0.03885 | 0.34732 | 0.03171 | 0.9679 | 28 | 28.2072 | 1.01453 | 35.5961 | 2.19798 | 0.1159 |
| **42** | 0.42411 | 0.02223 | 0.47143 | 0.02628 | 0.6874 | 42 | 28.4201 | 1.39846 | 36.6436 | 2.33341 | 0.1004 |

| **Serum ALT (U/L)** | | | | | |
|---|---|---|---|---|---|
| | **RBC** | | **PA** | | |
| **Day** | **Mean** | **SEM** | **Mean** | **SEM** | **p value** |
| **-6** | 11.6581 | 0.98838 | 11.4282 | 0.61796 | >0.9999 |
| **1** | 9.16728 | 0.25087 | 7.90693 | 2.22286 | 0.9901 |
| **15** | 8.59246 | 0.30922 | 8.00912 | 0.41975 | 0.83 |
| **28** | 7.30231 | 1.52722 | 9.53345 | 0.64389 | 0.7332 |
| **42** | 9.43551 | 0.61964 | 11.5432 | 0.78617 | 0.3051 |

Means ± SEM for hematology analysis conducted on plasma obtained by submandibular cheek bleeding from mice vaccinated with 1 μg RBC-STING or Pa-STING subcutaneously on days 0, 7, and 14. Plasma and serum were obtained on day -6, 1, 15, 28, and 42. Serum creatinine and aspartate aminotransferase (AST) were assessed by colorimetric kits according to the manufacturer's instructions. All other values were obtained by Hemavet analysis.

## REFERENCE


1. Van Hoecke L, Job ER, Saelens X, Roose K. Bronchoalveolar lavage of murine lungs to analyze inflammatory cell infiltration. *J Vis Exp*. 2017;(123):e55398.
