# Supplementary figures and images for: STING-adjuvanted outer membrane vesicle nanoparticle vaccine against Pseudomonas aeruginosa
Source: JCI Insight. 2025 Jul 24;10(17):e188105. doi: 10.1172/jci.insight.188105 (PMC12487677; doi:10.1172/jci.insight.188105)

UNCROPPED IMAGE FOR  
FIGURE 1G

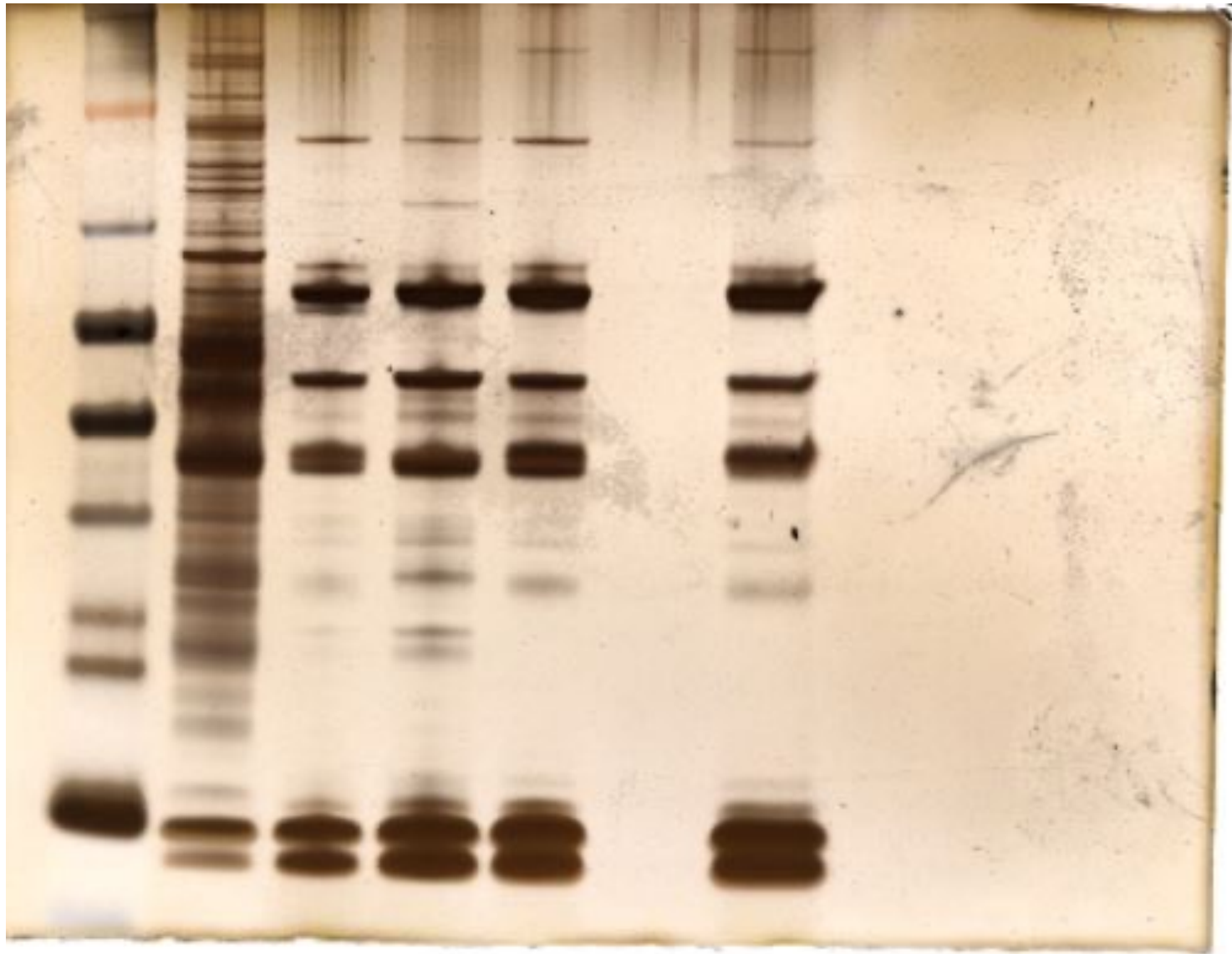

UNCROPPED IMAGE FOR  
SUPPLEMENTAL FIGURE 9B

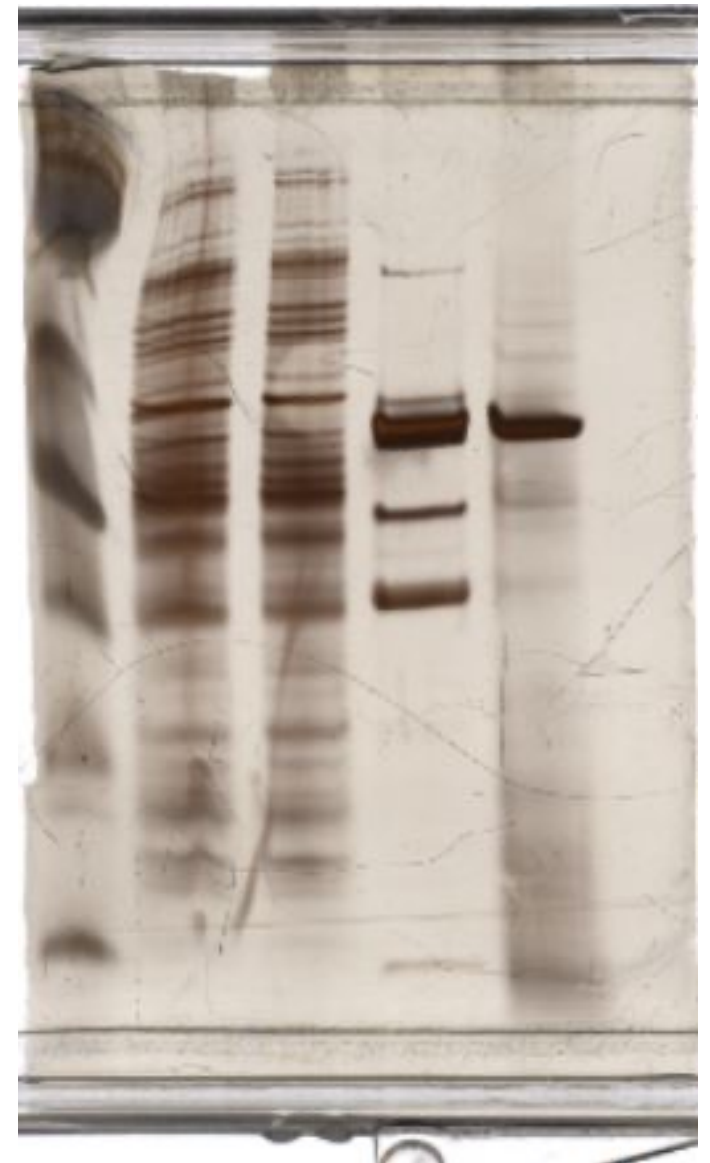

Supplement: Unedited blot and gel images [file jciinsight-10-188105-s237.pdf]
